# Phylogenetic Position of a Copper Age Sheep (*Ovis aries*) Mitochondrial DNA

**DOI:** 10.1371/journal.pone.0033792

**Published:** 2012-03-23

**Authors:** Cristina Olivieri, Luca Ermini, Ermanno Rizzi, Giorgio Corti, Stefania Luciani, Isolina Marota, Gianluca De Bellis, Franco Rollo

**Affiliations:** 1 Laboratorio di Archeo-Antropologia molecolare/DNA Antico, Scuola di Bioscienze e Biotecnologie, University of Camerino, Camerino, Italy; 2 Section of Haemato-Oncology, The Institute of Cancer Research, Sutton, United Kingdom; 3 Istituto di Tecnologie Biomediche, Consiglio Nazionale delle Ricerche, Segrate, Italy; Institut de Biologia Evolutiva - Universitat Pompeu Fabra, Spain

## Abstract

**Background:**

Sheep (*Ovis aries*) were domesticated in the Fertile Crescent region about 9,000-8,000 years ago. Currently, few mitochondrial (mt) DNA studies are available on archaeological sheep. In particular, no data on archaeological European sheep are available.

**Methodology/Principal Findings:**

Here we describe the first portion of mtDNA sequence of a Copper Age European sheep. DNA was extracted from hair shafts which were part of the clothes of the so-called Tyrolean Iceman or Ötzi (5,350 - 5,100 years before present). Mitochondrial DNA (a total of 2,429 base pairs, encompassing a portion of the control region, tRNA^Phe^, a portion of the 12S rRNA gene, and the whole cytochrome B gene) was sequenced using a mixed sequencing procedure based on PCR amplification and 454 sequencing of pooled amplification products. We have compared the sequence with the corresponding sequence of 334 extant lineages.

**Conclusions/Significance:**

A phylogenetic network based on a new cladistic notation for the mitochondrial diversity of domestic sheep shows that the Ötzi's sheep falls within haplogroup B, thus demonstrating that sheep belonging to this haplogroup were already present in the Alps more than 5,000 years ago. On the other hand, the lineage of the Ötzi's sheep is defined by two transitions (16147, and 16440) which, assembled together, define a motif that has not yet been identified in modern sheep populations.

## Introduction

Sheep (*Ovis aries*), have provided a farmed source of food, wool and hide since the Neolithic Agricultural period and represent one of the earliest to have been domesticated. Archaeozoological evidence suggests that sheep were probably first domesticated in the Fertile Crescent region in the Near East, around 9,000-8,000 years before present (b.p.) and spread out from the domestication centers in Europe, Asia, and Africa during the subsequent few thousand years [1].

Recent advances in genetics and the use of mitochondrial DNA (mtDNA) to characterize sheep genetic diversity are elucidating the origins of domestic sheep and their human-mediated global migrations. Studies performed on the control region fragment and/or the cytochrome b (*cytB*) gene of mtDNA of modern sheep from a wide geographical range describe five different haplogroups (A, B, C, D, and E) [2], [3], [4], [5], [6], [7], [8] into which the domestic sheep's genetic diversity divides. Haplogroup A and haplogroup B are the most frequent. These two haplogroups have been found in every geographic region where the domestic sheep have been sampled. In particular, haplogroup A is mainly represented in Asian breeds, while haplogroup B is found in high frequency in breeds sampled in Europe [2], [3]. Haplogroup C, on the other hand, is less frequent. Only few samples have been isolated in Asia, within the Fertile Crescent, and in Europe within the Caucasus and the Iberian Peninsula [5], [6], [8], [9], [10]. Haplogroup D and haplogroup E have been recently identified and, at the present are the rarest; sheep belonging to these two haplogroups have only been found in the Caucasus and Turkey [8], [9]. A recent study on the complete mitochondrial genome of ten domestic sheep and six wild sheep examined the relationship between domestic and wild sheep. The phylogenetic analysis confirms the division of domestic sheep into the five (A, B, C, D, E) haplogroups [11].

The development of technologies for the analysis of ancient DNA (aDNA) has opened a new field for inter-disciplinary work on documenting domestication. Ancient DNA sheds a more direct light on the process, potentially allowing a definitive identification of the particulars of domestication. Despite the relative abundance of reports on the analysis of mtDNA from modern sheep, at the present, little data are available for archaeological sheep [12], [13], [14] and in particular no data exist for European ancient sheep.

The most important archaeological discovery of the century was perhaps the finding of the mummified corpse of a prehistoric man, popularly known as the “Tyrolean Iceman” or “Ötzi”. The mummy was found at 3,270 m above sea level on the Alps, near the Austro-Italian border. Radiocarbon dating indicated an age between 5,350 and 5,100 years [15] corresponding to the Copper Age (Calcholithic). The mummy has been the object of a number of scientific investigations. Recently we have sequenced the whole mitochondrial genome of the mummy. We have found that it belongs to a K1 haplogroup branch (K1ö) not yet identified in modern populations [16].

A remarkable feature of this archaeological discovery is that the body was not found alone, but accompanied by the remains of clothes and equipment. Ötzi's clothes were mainly made of leather, and fur. They have been the object of scientific investigations since their discovery. Microscopic analysis indicated that the majority of the animal hairs are those of red deer (*Cervus*), while the remainders are goat hair [17]. In 1992, Lange [18] identified some parts of the clothing as tanned skin from chamois (Rupicapra). More recently, using a matrix-assisted laser desorption/ionization time-of-flight mass spectrometric (MALDI-TOF MS)-based analytical method, two samples from Ötzi's fur garments and one sample of his legging were assigned to sheep, while the upper leather of his shoes was assigned to cattle [19].

Here we report the sequencing of two mtDNA fragments of sheep (*Ovis aries*) identified from the genetic analysis of black animal hair shafts which were part of the Ötzi's clothes. Hairs have been preserved in glacier ice at an average temperature of about −10°C. These conditions have previously been shown to preserve the DNA of the mummy [16] and of associated animal and plant remains [20]. The Copper Age sheep sequences have been determined using massive sequencing of pooled amplification products by 454/Roche Genome Sequencer; this method has already been proven highly effective in producing the complete sequence of the mummy mitochondrial genome [16]. In addition, the preservation degree of the hair DNA has been further assessed through the analysis of nucleotide misincorporations. Finally, phylogenetic analysis was also performed to test the consistency of the sequences obtained and to explore the relationship between the Copper Age sheep mtDNA and the modern sheep mtDNA. Currently, a clear nomenclature for clustering domestic sheep mitochondrial sequences based on signature mutations along different branches is missing. In order to fill this gap, here we propose a cladistic signature notation based on world-wide mitochondrial control region-cytochrome B (*cytB*) domestic sheep sequence diversity (334 sequences available in Gen Bank).

## Results

### Sequencing of the Copper Age sheep mtDNA

DNA was successfully extracted from hair shafts collected from Ötzi's fur clothing. To identify the species to which the hairs belong, the DNA extracted was PCR amplified using the MBos L1269/MBos H1346 universal oligonucleotide primer pair for mammal DNA (Table S1) [20]. The system is designed to bind to an approximately 117 base pair (bp) long fragment of the mtDNA 12S ribosomal RNA gene. The amplification product was then cloned and four clones were analyzed by Sanger sequencing. The consensus sequence was compared with the sequences deposited in GenBank by using a BLAST search. The sequence corresponded 100% to that of sheep (*Ovis aries*).

Following species identification, the DNA extracted was PCR amplified using two sets of 15 and 13 oligonucleotide primer pairs (Table S1), respectively. The first set encompasses part of the mitochondrial control region, part of the 12S rRNA coding region (MT-RNR1) and tRNA^Phe^ (reference sequence NC001941 positions 15,983-592). The second primer set encompasses a 1,224 bp fragment of the complete *cytB* gene. All the 28 amplifications gave positive results. The products were diluted to equal concentrations, pooled and used as a substrate to prepare a library for sequencing using a GS-454/Roche Genome Sequencer (FLX Roche 454 LifeSciences). The sequencer yielded a total of 1,635 reads which were further processed by means of the GS Amplicon Variant Analyzer application (Roche) resulting in 1,276 reads (∼78% of the total); 359 reads were discarded because they either lacking one or both primers (179 reads) or were shorter than 50 bp (primer dimers, 15 reads) or were longer than 50 bp but showed no specific match or no match at all when used to scan the NCBI database (165 reads). Processed reads were assigned to 28 distinct clonal groups, which were assembled together on the basis of the overlapping tracts and their position in relation to sheep reference mtDNA sequence (NC001941). The 28 clonal groups showed a coverage ranging from 8 to 124 reads, corresponding to a mean coverage of about 45 and a median coverage of about 40 reads. During the assembly step of the reads, it was noted that the Ovis aries L16570/Ovis aries H60 amplification system was represented by three non complete reads only. For this reason, the PCR product was cloned into a plasmid vector and 10 clones were sequenced with conventional Sanger technology.

As a control, the 28 PCR products were sequenced also with conventional Sanger technology. Given that the 454 sequencing does not efficiently processes indels and homopolymeric regions, these were the object of a particularly accurate scrutiny [21], [22], [23]. In particular three indels (2 insertion and 1 deletion) were found in the sequences obtained by Sanger sequencing (Ovis aries L484/Ovis aries H592, Ovis aries L16221/Ovis aries H16386 and Ovis aries L16378/Ovis aries 16499), while 454 sequencing gave ambiguous results. In this case the indels were confirmed by a further PCR amplification and Sanger sequencing.

The polymorphisms in the Copper Age sheep obtained by the two methods (454 and Sanger sequencing) compared to NC001941 are reported in Table 1. We can observe that all the polymorphisms are in the “mtCR” region. On the other hand the *cytB* fragment does not show differences with the reference sequence (Table 1).

**Table 1:** Copper Age sheep nucleotide polymorphisms relative to *Ovis aries* reference sequence (NC001941).

Nucleotide position	Reference sequence (NC001941)	Copper Age Sheep
16128	C	T^(454)(S)^
16173^×^	T	C/T^(454)^;T^(S)^
16147	T	C^(454)(S)^
16343	T	C^(454)(S)^
16343 ins		C^(454)(S)^
16353^×^	C	T^(454)^;C^(S)^
16440	T	C^(454)(S)^
16472	T	del-T ^(454)(S)^
281	T	C^(454)^ ^(S)^
565 ins		G^(454)(S)^

^(454)^: nucleotide polymorphism analyzed by 454 technology; ^(S)^: nucleotide polymorphism variations analyzed by Sanger technology; ^×^: ambiguous nucleotide position.

The comparison between the reads obtained by the 454/Roche Genome Sequencer and the sequences achieved by direct conventional Sanger sequencing shows the presence of two ambiguous nucleotides at positions 16173 and 16353.

The nucleotide position 16173 was amplified by Ovis aries L16154/Ovis aries H16267 and Ovis aries L16119/Ovis aries H16182 amplification systems. In the three Sanger sequences (two sequences of Ovis aries L16154/Ovis aries H16267 and one sequence of Ovis aries L16119/Ovis aries H16182), this position unambiguously showed a thymine. All 454 reads corresponding to Ovis aries L16119/Ovis aries H16182 amplification system showed a thymine, but only half reads obtained from the Ovis aries L16154/Ovis aries H16267 amplification system showed a thymine, the remaining half showed a cytosine. Taking into account that, all the three Sanger sequences showed a thymine at position 16173, and that a cytosine at the same nucleotide position has been never described in modern sheep, in the final mtDNA Copper Age sheep a thymine was used.

The 16353 nucleotide position was determined using Ovis aries L16221/Ovis aries H16386 amplification system. The sequences obtained by 454 technology showed a thymine in this position. However, a thymine in position 16353 was never described in modern sheep. In order to resolve this issue, we have checked this nucleotide position using two independent PCR amplifications followed by direct sequencing. In both cases we found a cytosine in position 16353. We therefore decided to use a 16353 cytosine in the Copper Age sheep sequence.

### Nucleotide misincorporation analysis

**Figure 1:**
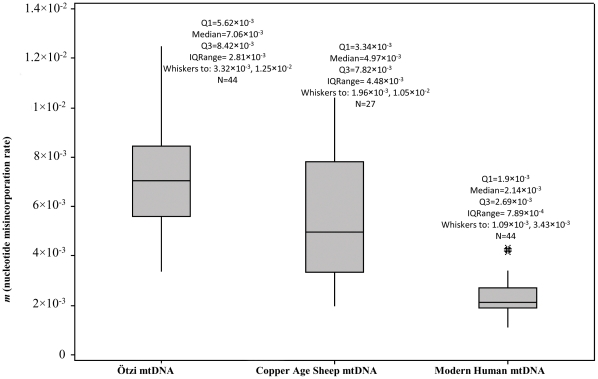
Nucleotide misincorporation rate (*m*) in Copper Age sheep, Copper Age human (Ötzi) and modern human mtDNA. The plot comprises a box and whiskers. A line is drawn across the box to represent the median; the bottom of the box is the first quartile (Q1) and the top is the third quartile (Q3). The lower whisker extends to the lowest value within the lower limit, while the upper whisker extends to the highest value within the upper limit. The limits are defined by: Q1+1.5 (Q3-Q1) (lower limit) and Q3+1.5 (Q3-Q1) (upper limit). The asterisk represent the outlier, a value beyond the whiskers.

Statistical analysis was applied to compare the nucleotide misincorporation values calculated in the Copper Age sheep, Ötzi and modern mtDNA sequences. In Table S2 are reported the nucleotide misincorporation rates (*m*) observed in each 454 clonal group analyzed for the Copper Age mtDNA sequences. The Two Sample T-Tests and non-parametric Mann-Whitney tests showed that the null hypothesis (H_0_) can be rejected when Copper Age sheep mtDNA reads are compared both with modern human mtDNA reads [24] and with Ötzi's mtDNA sequences [16], [24] (Table S3). The box-plot (Figure 1), employed to compare the *m* values in the sheep mtDNA sequences to the values obtained in the Ötzi's and modern mtDNA sequences [24], demonstrate that the *m* value variability in the sheep sequences is far from the *m* values in modern sequences and closer to the variability found in Ötzi's sequences. In particular, the mean and median *m* values in the sheep sequences (5.47×10^−3^ and 4.97×10^−3^, respectively) are lower than the values noted in the Ötzi's sequences (mean: 7.26×10^−3^, median: 7.06×10^−3^).

Taking into account type 1 and type 2 nucleotide misincorporations in the Copper Age sheep sequences we observed an overrepresentation of the type 2 (type 1∶type 2 ratio ∼1∶4). An analogous state was remarked when individual 454 clonal groups were considered. The Two Sample T-Tests and non-parametric Mann-Whitney tests were performed to accept or reject the H_0_ hypothesis that the number of type 1 and type 2 transitions in the sheep, Ötzi and modern human mtDNA sequences are the same. In the case of type 1, the tests support the H_0_ hypothesis for the Copper Age Sheep and Ötzi mtDNA sequences and reject the null hypothesis for the comparison between Copper Age Sheep and modern human mtDNA sequences (Table S4A).

**Figure 2:**
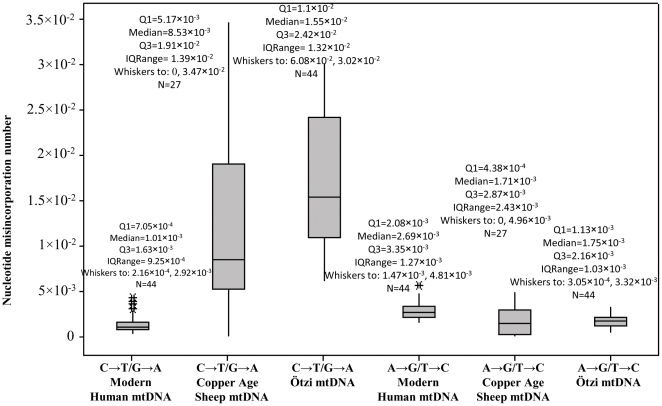
Nucleotide misincorporations number for type 1 and type 2 transitions in Copper Age sheep, Copper Age human (Ötzi) and modern human mtDNA. The plot comprises a box and whiskers. A line is drawn across the box to represent the median; the bottom of the box is the first quartile (Q1) and the top is the third quartile (Q3). The lower whisker extends to the lowest value within the lower limit, while the upper whisker extends to the highest value within the upper limit. The limits are defined by: Q1+1.5 (Q3-Q1) (lower limit) and Q3+1.5 (Q3-Q1) (upper limit). The asterisk represent the outlier, a value beyond the whiskers.

Regarding type 2 transitions the H_0_ hypothesis is rejected both in the comparison involving Copper Age sheep and modern human mtDNA sequences, and in the statistical tests between Copper Age sheep and Ötzi mtDNA sequences. The results of the tests are reported in Table S4B. The box-plot graph in Figure 2 displays the variability of type 1 and type 2 transitions in the Copper Age sheep mtDNA, Ötzi mtDNA and in the modern human mtDNA sequences. Clearly, the bigger difference between the three samples is represented by type 2 transition. The variability of the type 2 transitions in the sheep sequences is highly different from the values showed in the modern human mtDNA, alternatively is close to the values found in the Ötzi mtDNA sequences. In detail, the mean and median of type 2 values in the sheep sequences (1.19×10^−2^ and 8.53×10^−2^, respectively) are lower than the values marked in the Ötzi's sequences (mean: 1.70×10^−2^, median: 1.55×10^−2^).

### A portrait of modern domestic sheep phylogeny

We used a combined mtCR-*cytB* region (2027 bp) to describe the phylogenetic relationships of modern domestic sheep (*Ovis aries*) and to infer the phylogenetic position of the Copper Age sheep. For this purpose we retrieved from Gene Bank 335 published world-wide mtCR-*cytB* sequences (Table S5) and determined the mtDNA sequence of an ancient sheep. Our analysis detected a total number of 210 segregating sites characterizing 161 haplotypes.

We inferred a median joining network by making use of sequence variation and by placing an *Ovis vignei* mtDNA as outgroup. The inferred network showed 374 substitutions with 333 transitions and 41 transversions. Two nucleotide transitions at positions 16440 and 16453 occurred with the highest frequency within the network and can be reported as the fastest sites or hotspots (Figure S1).

**Figure 3:**
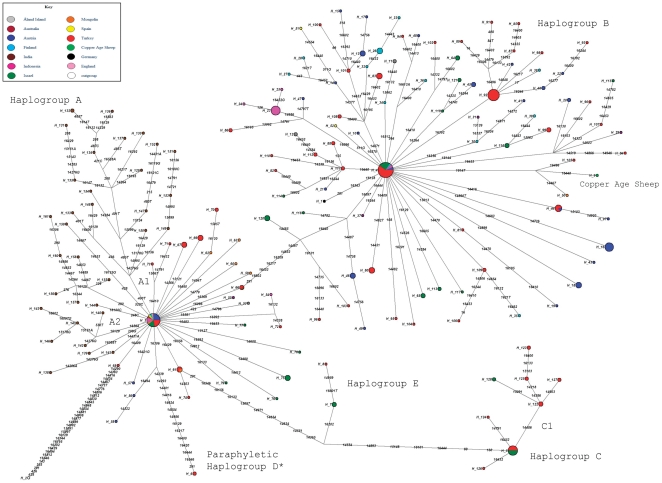
Median-joining phylogenetic network of modern sheep based on 336 sequences and rooted with *Ovis vignei* (H_2). Each cluster symbolized by a circle refers to a haplogroup. Line connecting each circle represents phylogenetic branches. Numbers along each branch are transitions and refer to nucleotide positions variants relative to *Ovis aries* reference sequence (NC_001941). * paraphyletic group.

The topology of our world-wide mtDNA network based on 336 sequences matched published trees [7] in most respects, with certain minor exceptions of details (Figure 3). The network identified mutations along different branches which provide motifs, or signature polymorphisms, for clustering different branches as suggested by Richards et al. [25]. Consequently, here we introduce a new cladistic notation for the worldwide mitochondrial diversity of domestic sheep. We define a mtDNA cluster within the network as a group of at least three different haplotypes sharing a motif (at least two signature polymorphisms) or a single signature polymorphism which occurs only once within the world-wide network. For sake of simplicity, we apply this criterion of classification only to clusters budding from the predominant haplotype of each haplogroup. We only make use of this criterion for clusters showing a coherent geographic or breed distribution. Our nomenclature also refers to Meadows et al. [7] who analysed combined 197 mtCR-*cytB* sequences in domestic sheep and reported five haplogroups termed A, B, C, D and E. We refer to paraphyletic collection of mtDNAs outside these five main clades with a star letter (i.e. A*).

Our phylogenetic network shows that 90% of the mtDNA pool can be clearly grouped into two big clades referred as haplogroup A and haplogroup B.

Haplogoup A shows a predominant haplotype (H_3) from which a star-like phylogeny departs. This haplotype contains 24% of the haplogroup A mtDNA pool. One large subclade within the cluster can be assigned by the transversion 400A→T and includes 17 haplotypes of domestic sheep from India. We propose that this signature polymorphism defines a geographically defined subcluster of A, named sub-haplogroup A1. Within this subcluster the transversion 401A→T clusters only sheep of the bannur breed (Table S5). Another sub-cluster of sequences from India, A2, is defined by two transitions of the motif 16129–16564. This sub-cluster comprises only garole breed sheep (Table S5). Haplogroup A is defined by the motif 291, 14467, 14653, 16097, 16209, 16217, 16440, 16453 and 16602.

The *Ovis aries* reference sequence (NC001941) fall within the haplogroup B where the predominant haplotype H_4 incorporates about 15% of mtDNA pool classified within haplogroup B. A star-like phylogeny departs from this haplotype. According to our clustering criteria we may define two sub-clusters of haplogroup B, one characterised by the transition 16264, and one by the transition 364. Both polymorphisms happen only once in the world-wide network. However the hypothetical subcluster defined by 16264 comprises sheep from Austria (breed: tyrolean stone) and Israel (breed: awassi), two breeds geographically and morphologically distant and, for this reason, we prefer to mention it only as a feasible subcluster of B rather than to assign a name to it. For the same reason we only report the subcluster defined by 364 which comprises breeds from Turkey, Austria and Finland.

Six haplotypes H_120, H_118, H_96, H_103, H_46 and H_49 are placed within the branch connecting haplogroups A and B. Although we can not resolve the cluster of these haplotypes, they share 7 or 8 out of 9 signature polymorphisms with haplogroup B and, we decided to classify them as paraphyletic groups B* [25].

The network shows a branch leading to a super-cluster CE defined by the motif 291, 14365, 14551, 14634, 14854, 14971, 15097, 16133, 16156 and 16546.

Haplogroup C is characterised by the motif 69, 160, 14554, 14893, 15148, 16101 and 16444 and comprises 7% of the sequences within the network. Haplogroup C includes only sheep from Middle East. We can also define as subcluster C1 defined by the transition 14486.

Haplogroup E consists of only two haplotypes containing four sheep from Middle East and characterised by the transition 16202. This haplogroup should be clustered with caution as the small number of sequences included could be inadequate to define a haplogroup, but we prefer to delineate it as, already reported by Meadows et al. [7].

A long branch gemming from A haplogroup characterised by the motif 291, 14239, 14293, 14401, 14416, 14624, 14854, 14986, 16129, 16217, 16400, 16420, 16444 and 16546 leads to the haplotype H_6. Two sequences (Table S5) reported as cluster haplogroup D [7], make this haplotype. As, at the present, there is only one small in size haplotype within this cluster, we prefer to label it as paraphyletic haplogroup D*. The nucleotide position 291 showed along the D* branch and also in the lineage towards the super-cluster CE is a reverse transition T→C which restore the nucleotide carried by the *Ovis aries* reference sequence.

A schematic representation of the sheep phylogenetic network is shown in Figure S2 while the detailed world-wide sheep phylogeny is displayed in Figure S3.

In order to delineate the geographical distribution of each haplogroup we clustered the samples in four main world-wide regions, Southern Central Asia, Europe, Middle East and Oceania. No mtCR-*cytB* sequences from Africa or America have been found in the scientific literature. The frequencies and geographical distribution of each haplogroup are reported in Table S6 and Figure S3 respectively. Haplogroup A is the most representative cluster in Southern Central Asia while haplogroup B is the most frequent within Europe and Oceania. The Middle East on the other hand, shows the presence of all inferred haplogroups. The haplogroup frequencies in Oceania may be under estimated due the small number of sequences considered (Table S5).

### The ancient sheep sequence within a modern phylogenetic network

To test the consistency of the Copper Age sheep mtDNA assembly and to determine the phylogenetic position, we placed the ancient mtCR-*cytB* sequence in the inferred phylogenetic network [16], [26]. The Copper Age sheep's sequence falls within haplogroup B with all the mutational-motif positions that identify this haplogroup (Figure 3 and Table 1). The Copper Age sheep's sequence shares a transition at position 16147 with two single sequences belonging to a Tibetan breed sheep from Mongolia (Genbank number: AY879433/AY879554; H_50) and to a Turkish Cine Capari breed (Genbank numbers; DQ852187/DQ851990; H_105). The transition 16440 leading to the Copper Age sheep is one of two most frequent transitions of the network (Figure S1) and can not be regarded as a transition that defines a lineage.

## Discussion

### Authenticity and accuracy of the Copper Age sheep mtDNA sequences

It has been shown that the hairs, compared to other samples (such as bone and teeth), are largely resistant to penetration by contaminant DNA during the handling by human [27]. Therefore the hairs represent a more reliable and more contamination-resistant tissue for use in ancient DNA analyses than others. In our case we have animal (sheep) hairs that have been preserved for approximately 5,000 years under glacier ice at an average temperature of about −10°C. These conditions have been previously shown to preserve the DNA of the mummy and of associated animal and plant remains [16], [20]. In the case of the present study, therefore, contamination is probably not a problem, but we have also adopted strict laboratory conditions to ensure the authenticity of our results as reported in Materials and Methods.

- The Copper Age sheep sequences have been determined using massive sequencing of pooled amplification products using 454/Roche Genome Sequencer. The power of this method has been demonstrated in the analysis of the Ötzi's complete mitochondrial genome [16].
- The consensus mtDNA sequences obtained through the massive sequencing have been compared with the sequences obtained by direct Sanger sequencing. Ambiguous nucleotide positions detected during the comparison have been elucidated by performing several independent PCR amplifications followed by direct Sanger sequencing. In particular, in the case of the amplification system Ovis aries L16570/Ovis aries H60 we cloned the amplification products in a plasmid and sequenced several clones using Sanger technology. This approach allowed us to overcome the low coverage obtained by the 454 platform for this sequence.
- The 454 sequencing does not efficiently processes indels and homopolymeric regions [21], [22], [23] and these were object of an accurate scrutiny. In particular, to confirm three indels detected with 454, the relevant DNA segments were PCR amplified and Sanger sequenced twice (Table 1).
- The authenticity of the sequences obtained was further tested by analyzing the nucleotide misincorporation rate. This analysis has shown that the sequences obtained through massive sequencing are characterized by an higher number of type 2 transitions compared to type 1 transitions (Figure 2). This result is consistent with the post-mortem pattern of cytosine deamination that characterizes the ancient DNA sequences [24].

In conclusion, the “*modus operandi*” implemented in our work guarantees that the Copper Age sheep mtDNA sequences obtained are authentic and accurate. The excellent preservation conditions of the specimen and the strict laboratory procedures employed, make extremely unlikely the contamination by modern sheep DNA. Consequently, we think that additional controls like multiple DNA extractions and replication of the results in another laboratory are unnecessary.

### The Copper Age sheep mtDNA preservation issue

Several studies have investigated the possibility of analyzing the DNA extracted from ancient hair shafts. Mitochondrial DNA has been successfully extracted from old hair samples, including ∼64,800 year-old bison [28], Siberian mammoths dating from ∼46,000 and ∼18,500 years BP [29], a ∼4,000-year-old extinct Palaeo-Eskimo [30], 100-years-old Native Americans [31]. The relevance of hair shafts, in the aDNA studies, is linked to the minor susceptibility of these samples to contamination by exogenous sources of DNA. This resistance to the incorporation of contaminant DNA could be related to the hydrophobic nature of the proteins filling hair cuticle cells and to the impermeable nature of the keratin structures forming the hair shaft [32].

In this context, hairs shafts collected from the Ötzi's clothing, represent an excellent starting point to carry out molecular studies. Furthermore, the environmental history and preservation of the sample in the ice until its discovery [33] could suggest a good degree of DNA conservation. A preliminary DNA amplification, using mammal universal primers, and the subsequent sequencing of the 12S mtDNA hair shafts, for the purpose of identifying the species belonging to the sample, have shown that the extracted DNA matched the sheep (*Ovis aries*) genetic material. It is extremely interesting to note that all the clones contained fragments of the sheep mtDNA and none of them contained contaminant human DNA.

The state of DNA preservation in the hair shafts sample has been also assessed by analysis of the nucleotide misincorporation rate (*m*) and the extent of the type 1 and type 2 nucleotide misincorporations in the reads achieved following the 454 sequencing of the PCR products. In particular, the DNA damage has been evaluated comparing the mtDNA sequences from hairs shafts with other sequences (mummy mtDNA sequences and modern human mtDNA sequences) taken from the literature [24]. The nucleotide misincorporation rates (*m*), dependent on DNA damage and on polymerase error, in the Copper Age sheep mtDNA sequences are lower than those found for the Ötzi mtDNA sequences [16], [24] and obviously, higher than those found for modern human mtDNA [24], where the DNA damage is negligible. Going in detail, concerning the type 1 transitions, most likely due to the errors of the polymerase, the statistical analysis demonstrates that there is no statistically important difference in the amount of type 1 in the three samples: Copper Age sheep mtDNA, Ötzi mtDNA and modern human mtDNA sequences. Consequently, the lower *m* rates in the sheep mtDNA compared with the Ötzi mtDNA data seem to be attributable to the lower incidence of the type 2 transitions in the sheep mtDNA and therefore to the lower incidence of the cytosine hydrolytic deamination in the sheep mtDNA, that represents the main cause of miscoding lesions in ancient samples [24], [34]. The relatively low levels of hydrolytic damage induced lesions among sheep mtDNA sequences could be explained by the low quantities of free water associated with the keratin-packed hair cells that may also reduce hydrolytic damage of the DNA [28].

The excellent DNA preservation in the sheep hair shafts and the absence of contaminant DNA could be finally explained taking into account the Ötzi clothing tanning process. In 2010 a study performed on the Ötzi's leather clothes has highlighted a high concentration of calcium salts of saturated fatty acids. The researchers concluded that tanning of the Iceman's clothes partly consisted of saponified land animal fats, which had been physically incorporated, mainly in the form of calcium stearate [35]. The saponification process possibly generated a superior water-repellent effect on the leather and fur of the Ötzi's clothing giving rise to a protective shield against the contaminations and the hydrolytic damage of the DNA.

To conclude, our data confirm that the hair shafts represents an excellent start point to carry out genetic analysis. Moreover, the statistic analysis on the nucleotide misincorporations show that, although the two samples used to obtain the sheep and Ötzi mtDNA sequences have been preserved until the discovery at the same condition, the mtDNA preserved in the hair shafts sample is less susceptible to damage, in particular to cytosine hydrolytic damage.

### Phylogenetic analysis

Sheep were domesticated in the Fertile Crescent region in the Near East, around 9,000-8,000 years BP and spread out of the domestication centers in Europe, Asia, and Africa during the next few thousand years. After a very long period of soft selection, about two hundred years ago - with the introduction of the breed concept - the sheep have been subjected to stronger selection pressures. In particular, autochthonous breeds in some areas have been replaced with more competitive breeds and highly productive breeds have been selected for their reproductive traits. As a consequence, the selection pressures imposed by humans gave rise to the disappearance of many locally adapted breeds and, to some extent, to the loss of the genetic background [36].

The sheep analyzed in this study, the oldest sheep sample from Europe analysed until now, dated back about 5,350-5,100 years ago, did not undergo the later selection pressures, thus the genetic background is still preserved and available to provide information about the domestication history of the sheep.

To evaluate the relationship between the Copper Age sheep and modern domestic sheep, we have reconstructed a world-wide sheep phylogenetic network based on 334 mtCR-*cytB* sequences, and formulated a new cladistic notation for the mitochondrial diversity of domestic sheep. Our network attests to the branching structure of the sheep mtDNA genealogy, according to previously published trees [7] and shows a geographically robustness as sheep geographically close are clustered together. Few branches show a different degree of geographical bias when sheep coming from different locations are grouped together. This bias may be due to an undefined degree of homoplasy [37], sequencing errors [38] or a long history of sheep domestication, breeding and human migrations.

The phylogenetic network constructed represents all the haplogroups defined nowadays in the world-wide modern sheep and, it may be a useful reference for application in future studies dealing with mtDNA of domestic sheep. Further investigations using the complete sequencing of modern and ancient sheep mtDNA genomes should, in the future, allow researchers to enhance our clustering criteria and broaden the sheep phylogenetic diversity and haplogroups classification with considerable precision.

The phylogenetic analysis of the Copper Age sheep places the sample within haplogroup B, that represents the main haplogroup in the modern European sheep. The phylogenetic position of the Copper Age sheep strengthens its authenticity as the sequence bears all mutations defining the haplogroup B and does not show any phantom mutations or mosaic structure [26], [39]. The lineage of the Copper Age sheep is defined by two transitions 16147 and 16440 that together define a motif. The 16147 transition is not rare but may be geographically localised to the Asian continent as, at present, no modern European sheep show this polymorphism. The Copper Age sequence is the only European sheep carrying this polymorphism which may have arisen as a solitary mutation along the branch. We may speculate that the polymorphism 16147 together with the transition 16440 may indicate an ancient European branch that has disappeared over the time or perhaps, may refer to a very rare motif still not found within modern domestic sheep.

## Materials and Methods

### Ancient-DNA Work

We performed all manipulations of ancient-DNA sample at the Laboratorio di Archeo-Antropologia Molecolare/DNA Antico of the Camerino University, in a dedicated facility into which no modern animal DNA has ever been introduced. This facility is composed of an antechamber, in which the operator wears a full-body sterile suit, gloves, a face screen, a breathing mask, and a laboratory. Both environments are equipped with UV lights and a positive-pressure air-filtering system providing 99.97% particle elimination and a complete change of air every 10 min. We frequently clean all surfaces with bleach.

### Sample collection

Remains of the Ötzi 's clothing were recovered during the 1991 and 1992 archaeological explorations of the mummy site. In 1992 one of us (F.R.) obtained small specimens (in the range of approximately 100–200 mg each), from Lorenzo dal Ri, director of the archaeological superintendency of Bolzano, Italy, to perform DNA analyses. No further authorization was required. Since then the specimens have been kept in our laboratory at −20°C.

### DNA extraction

For DNA extraction we utilized 35 mg of black hair shafts which were part of the specimens obtained in 1992. The hairs were cut into small (∼0.5 cm) fragments. Subsequently the sample was manually washed several times with sterile H_2_O to remove any mud, and debris from the outside of the hairs. Digestion of the hairs was performed overnight at 55°C with rotation, using 600 µl of following digestion buffer: 10 mM Tris-HCl (pH 8.00), 10 mM NaCl, 2% w/v SDS, 5 mM CaCl, 2,5 mM EDTA (pH 8.00), 40 mM dithiothreitol (DTT; Cleland's reagent) and 10% proteinase K solution (>600 Mau/ml, Qiagen) [29]. After a lysis phase, the DNA was extracted using a phenol-chloroform protocol. The DNA fraction was precipitated from the final supernatant by centrifugation at 13,500 g for 5 min after the addition of 1/10 volume of 2 M sodium acetate and 2.5 volumes of cold (−20°C) ethanol. Finally, the DNA precipitates were re-suspended in 20 µl of sterile distilled water and stored at −80°C until use. We prepared extraction blanks throughout the procedure.

### Primer Design, PCR Amplification and mtDNA sequencing

Three regions of the mitochondrial DNA (mtDNA) genome were sequenced. The first region, corresponding to a fragment of the 12S ribosomal RNA (rRNA) gene (12SrDNA), was generated using PCR system for mammal DNA (Mbos L1269/Mbos H1346) that bind to a fragment of 117 bp in length (calculated on the basis of the *Bos taurus* sequence) [20]. The second region match and with a 1,204 bp fragment encompassing part of the mitochondrial control region (mtCR), part of the 12S rRNA coding region (MT-RNR1) and tRNA*^Phe^* (reference sequence NC001941 positions 16,004-592; in this paper this region will be referred to as “mtCR”). Finally the third region corresponded to a 1,225 bp fragment of the *Ovis aries* cytochrome b (*cyt b*) (NC001941 positions 14,103–15,327). Two sets of overlapping primers were designed from reference sequence to amplify these two last regions. In particular, sixteen and thirteen primer pairs were used to cover the two regions.

To design the primers we aligned different published sequences of *Ovis aries* in the Gene Bank. We selected primers located in the most conservative regions in order to keep the number of polymorphic positions in the oligonucleotides to a minimum. The PCR systems were directly tested on the ancient-DNA template, and no positive (i.e., modern DNA) control was used, to minimize the risk of contamination.

We performed DNA amplifications in 100 µl of reaction mix of the following composition: 10 mM Tris-HCl (pH 8.3), 50 mM KCl, 2.5 mM MgCl2, 2.5 enzyme units of Taq polymerase (HotStarTaq DNA polymerase, QIAGEN), 200 mM each dNTP, 300 ng each primer, and 1 µl of DNA template (diluted 1∶40 to reduce the effect of Taq polymerase inhibitors). We pretreated the reaction mixture with DNase (2 enzyme units for 30 min at room temperature) to eliminate any contaminant DNA and subsequently inactivated the DNase by heating to 95°C for 15 min. The thermal profile was as follows: 1 min at 95°C, 50 s at the relevant annealing temperature, and 1 min at 72°C, with a final extension of 10 min at 72°C. The number of cycles ranged from 45 to 55. To reduce the extent of errors generated by the reaction, we never reamplified any PCR product; only PCR products obtained after a first amplification round were used for the subsequent analysis.

The list of oligonucleotide primer-pairs utilized and the corresponding annealing temperatures are given in Table S1.

Amplification products were checked by electrophoresis on 2% (w/v) agarose, purified using the High Pure PCR Product purification kit (Roche Molecular Biochemicals, Mannheim, Germany). The products of the Mbos L1269/Mbos H1346 and Ovis aries L16570/Ovis aries H60 amplification systems were cloned, using the pGEM-T Easy Vector System (Promega Corp., Madison, WI) and the recombinant plasmids were isolated using a Miniprep kit (Promega, Madison, WI). We assessed insert size and DNA concentration using gel electrophoresis. The other PRC products were direct sequenced. DNA sequences were obtained using an ABI-Prism 310 automated DNA sequencer in the BMR-Genomics sequencing service (University of Padua). The sequences were checked and analyzed by Sequence Scanner version 1.0 (Applied Biosystems, Foster City, CA), and aligned with the program BioEdit v.7.0.9.

### 454 sample preparation and 454 platform sequencing

The amplification products were diluted to equal concentrations, then pooled in equimolar proportion and used as a substrate for the pyrosequencing reaction 16,40. We further spectrophotometrically quantitated and purified the mixture (MinElute PCR Purification Kit, QIAGEN, Hilden, Germany), and we checked the fragments length using the Agilent 2100 Bioanalyzer (Agilent Technologies, Palo Alto, CA, USA).. Sample was prepared as suggested by the 454/Roche GS FLX library preparation protocol. Because of the short size of the template, we did not perform any DNA fragmentation. The obtained sstDNA library was checked with Agilent 2100 Bioanalyzer and quantitated by RiboGreen RNA Quantitation Kit (Invitrogen, Carlsbad, CA). After emulsion PCR, we loaded the enriched beads onto 1/16^th^ of PicoTiterPlate, than the sample was sequenced using the 454/Roche Genome Sequencer. Reads were processed using GS Amplicon Variant Analyzer application (AVA) by Roche and .the resulting multi-alignments were then used to generate the consensus sequences by a home-made Python script, which assigned the most frequent base at each position.

### Nucleotide Misincorporation Analysis

The consensus sequence for each group of clones, produced by 454/Roche Genome Sequencer, was determined from the shared bases and the remaining interclone base differences (nucleotide misincorporation) were attributed to either post-mortem damage (miscoding lesions) or polymerase error. For each set of clones, the nucleotide misincorporation rate (*m*) was calculated in accordance with Olivieri et al. [24], and the number and type of nucleotide misincorporations were assessed according to Gilbert et al. [41]. Two-Sample T-Tests and non-parametric Mann-Whitney tests were performed to compare the nucleotide misincorporation rate (*m*) between: (a) Copper Age sheep mtDNA sequences and Ötzi mtDNA sequences [16]; (b) Copper Age sheep mtDNA sequences and modern human mtDNA sequences [24]. To highlight the distribution of *m* among the three groups of sequences, the *m* rates for each of them were represented using a box-plot. Similarly, Two-Sample T-Tests and non-parametric Mann-Whitney tests were used to compare the number of type 1 and type 2 transitions in the three groups of mtDNA sequences. Moreover, the values of these transitions were plotted to display their distribution. The statistical analysis was performed using the Minitab 15.1.0.0 software.

### Phylogenetic Analysis

The mtCR and *cytB* sequences obtained from Copper Age sheep were concatenated and aligned with the equivalent region of *Ovis aries* reference sequence (Gene bank accession number: NC001941). A full set of 334 *Ovis aries* sequences available in GenBank, and one sequence from *Ovis vignei* were also aligned with the ancient sequence control-region sequence using the ClustalW software implemented by the BioEdit program version 7.0.9 [42]. A phylogenetic network of the combined mtCR-*cytB* dataset of 336 sequences (Table S2) was constructed using Network 4.5 program (http://www.fluxus-technology.com), employing the median joining algorithm [43]. To reduce the probability of incorrect and inaccurate phylogenies in the network and faulty interpretations of global haplogroup relationships, sequence gaps (insertions and deletions in the alignment) were excluded from the analysis. In addition to achieve an easily readable network, nucleotide positions were weighted in inverse proportion to the number of mutations observed for each position. The *Ovis aries* reference sequence (Gene bank accession number: NC001941), was also used for the identification of nucleotide positions. In accordance with previously published works [7] we have drawn our network employing the median joining algorithm [43] instead of using the more robust reduced median [44]. We are aware of the fact that the former algorithm may over resolve the phylogeny and eliminate some reticulations of links that should be evaluated during the construction of the network.

## Supporting Information

Figure S1 **Hot Spots in the mtCR-** ***cyt B*** **region, showing all the positions that appear >4 times in the network.**
(TIFF)

Figure S2 **Schematic tree for world-wide sheep mitochondrial variation.** Each cluster symbolized by a circle refers to a haplogroup. Line connecting each circle represent phylogenetic branches. Dashed line describes a well a link leading to a paraphyletic group. Numbers along each branch are transitions and refer to nucleotide positions variants relative to *Ovis aries* reference sequence (NC_001941). * paraphyletic group.
(TIFF)

Figure S3 **Geographical distribution of haplogroups within four main geographic areas: Southern Central Asia, Europe, Middle East and Oceania.**
(TIFF)

Table S1 **Primer Systems Utilized, with the Corresponding Product Length and Annealing Temperature.**
(DOC)

Table S2 **Nucleotide misincorporation rate (m) within each Copper Age sheep mtDNA group of reads.**
(DOC)

Table S3 **Two Sample T-Tests and non-parametric Mann- Whitney tests between the values of** ***m*** **(nucleotide misincorporation rate) in Copper Age mtDNA, Ötzi mtDNA and modern human mtDNA.**
(DOC)

Table S4 **Two Sample T-Tests and non-parametric Mann- Whitney tests between the values of type 1 (A) and type 2 (B) in Copper Age sheep mtDNA, Ötzi mtDNA and modern human mtDNA.**
(DOC)

Table S5 **Sequences employed to infer the** ***Ovis aries*** **mtDNA phylogeny including the Copper Age sheep.**
(DOC)

Table S6 **Distribution of haplogroups (in percentage) within different geographic area.** The outgroup sequence *Ovis Vignei* is excluded. *Paraphyletic group.
(DOC)
