# Supplementary figures and images for: Phylogenetic Position of a Copper Age Sheep (Ovis aries) Mitochondrial DNA
Source: PLoS One. 2012 Mar 23;7(3):e33792. doi: 10.1371/journal.pone.0033792 (PMC3311544; doi:10.1371/journal.pone.0033792)

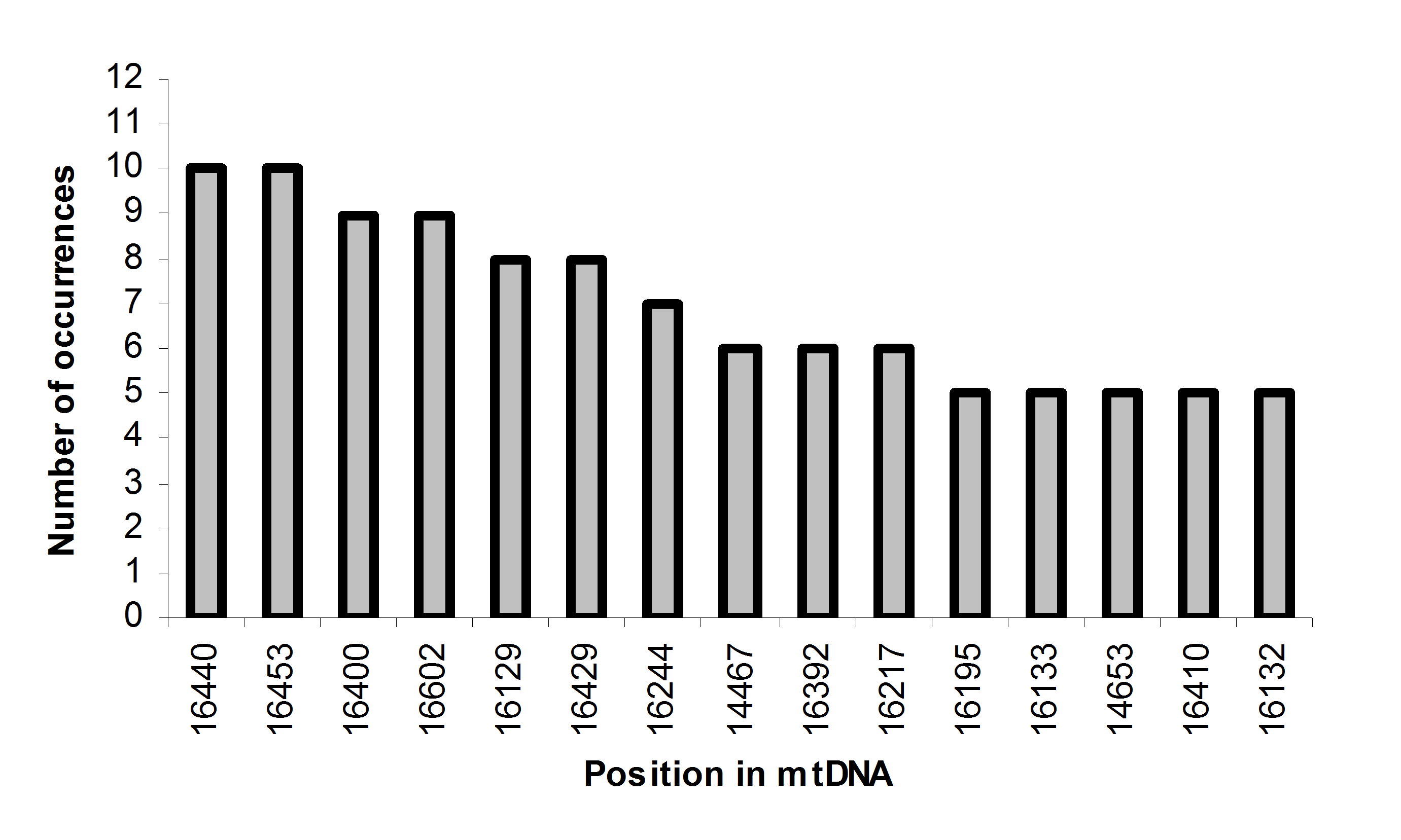

Supplement: Figure S1 — Hot Spots in the mtCR- cyt B region, showing all the positions that appear >4 times in the network. (TIFF) [file pone.0033792.s001.tiff]

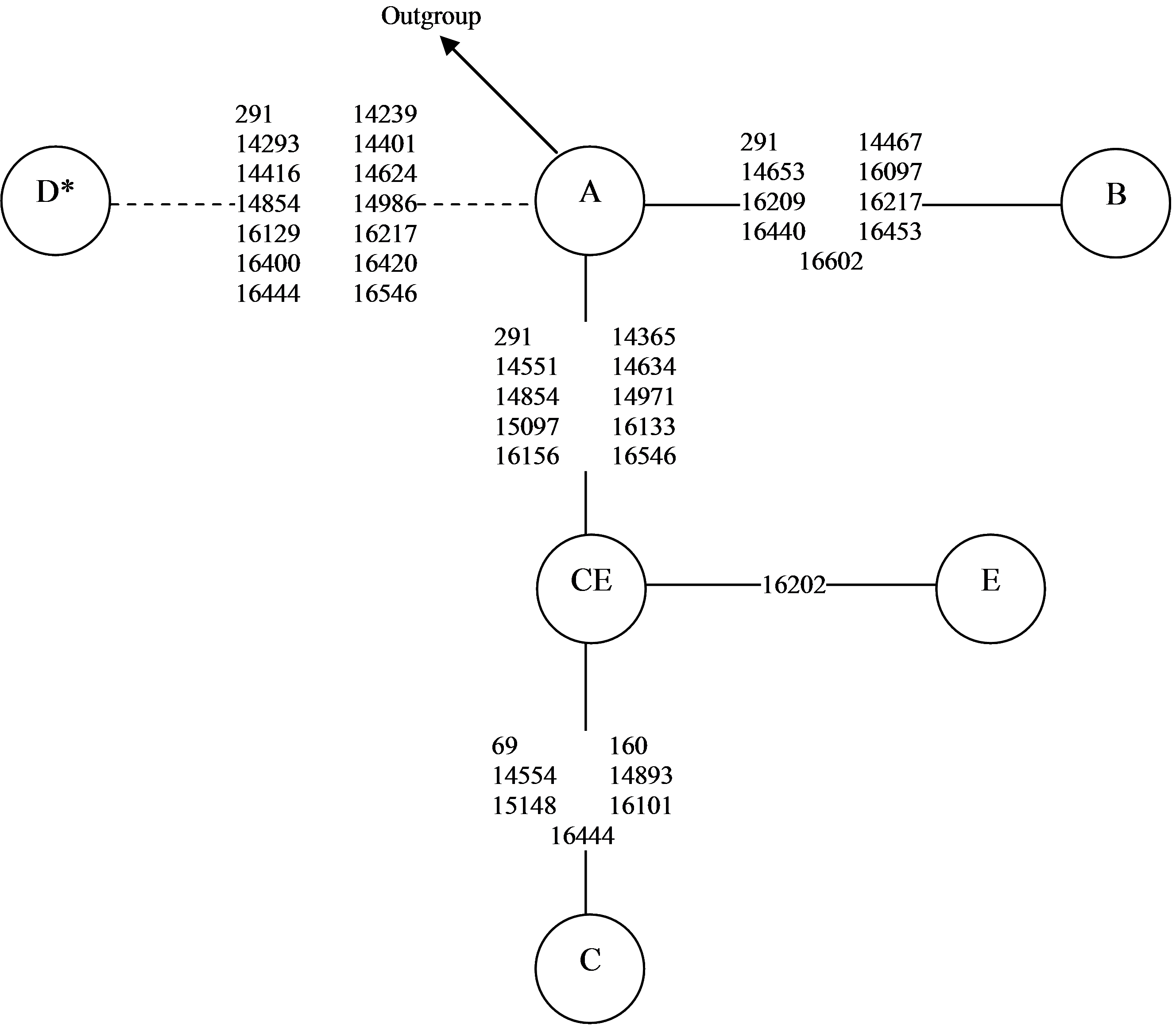

Supplement: Figure S2 — Schematic tree for world-wide sheep mitochondrial variation. Each cluster symbolized by a circle refers to a haplogroup. Line connecting each circle represent phylogenetic branches. Dashed line describes a well a link leading to a paraphyletic group. Numbers along each branch are transitions and refer to nucleotide positions variants relative to Ovis aries reference sequence (NC_001941). * paraphyletic group. (TIFF) [file pone.0033792.s002.tiff]

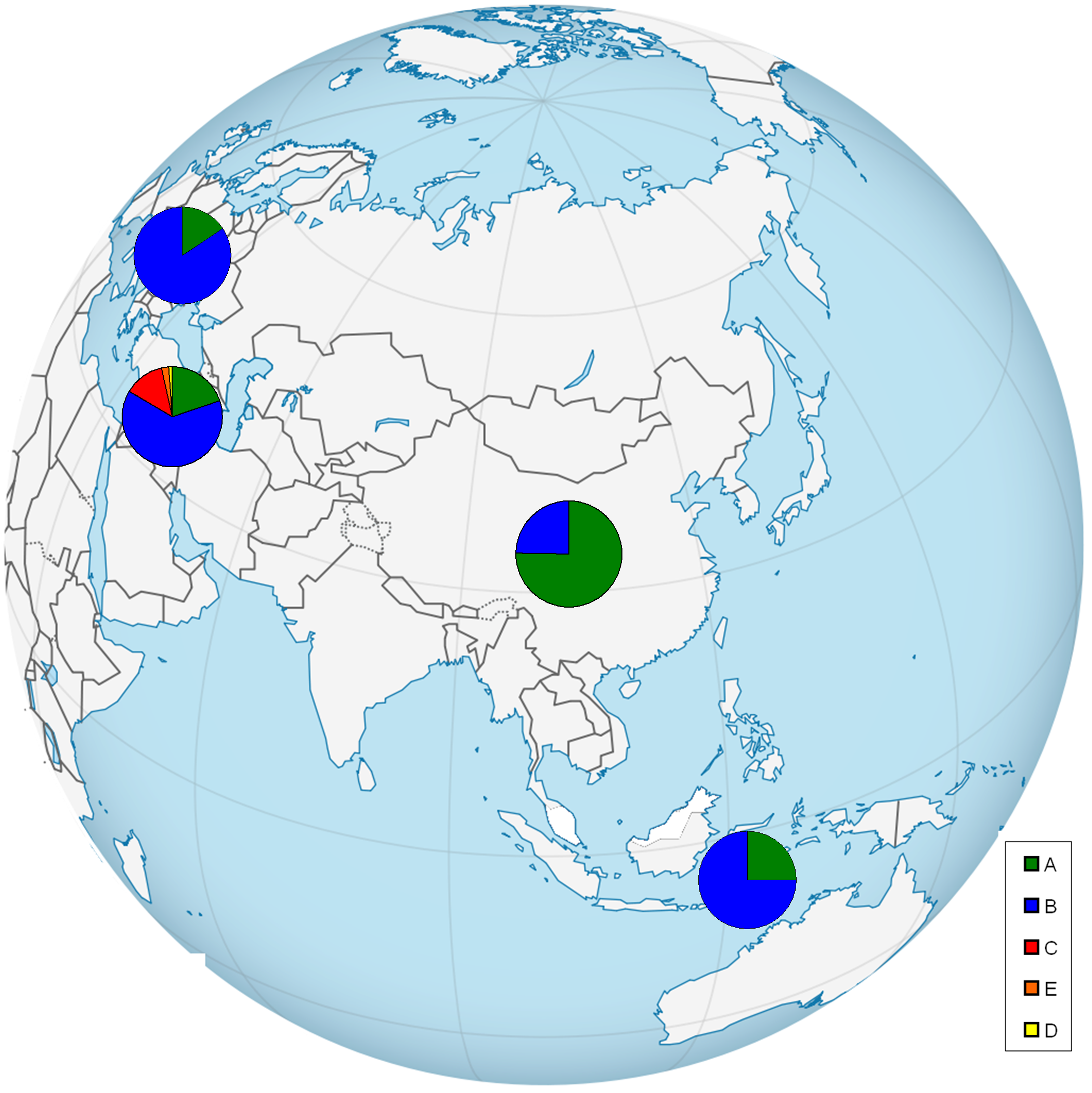

Supplement: Figure S3 — Geographical distribution of haplogroups within four main geographic areas: Southern Central Asia, Europe, Middle East and Oceania. (TIFF) [file pone.0033792.s003.tiff]
