# Supplementary material for: Phylogenetic Position of a Copper Age Sheep (Ovis aries) Mitochondrial DNA
Source: PLoS One. 2012 Mar 23;7(3):e33792. doi: 10.1371/journal.pone.0033792 (PMC3311544; doi:10.1371/journal.pone.0033792)
Supplement: Table S1 — Primer Systems Utilized, with the Corresponding Product Length and Annealing Temperature. (DOC) [file pone.0033792.s004.doc]

**Table S1.** Primer Systems Utilized, with the Corresponding Product Length and Annealing Temperature

| **Forvard** | **Sequence (5'-> 3')** | **Reverse** | **Sequence (5'-> 3')** | **Length (bp)** | **AT (°C)** |
| --- | --- | --- | --- | --- | --- |
| Mbos L1269 | CATGAAGCACGCACACACCG | Mbos H1346 | CCAGTATGCTTACCTTGTTAC | 117 | 50 |
| Ovis aries L16004 | AACTGCTTGACCGTACATAGTA | Ovis aries H16134 | TCATTAAATAGTTACCCCCAC | 172 | 52 |
| Ovis aries L16154 | TGTGGGGGTAACTATTTAATG | Ovis aries H16267 | ATGCATGACACCACAGTTATG | 154 | 52 |
| Ovis aries L16119 | AAGGATCCCTCTTCTCGCTC | Ovis aries H16182 | GATTTTAGATGAGATGGCCCTG | 104 | 58 |
| Ovis aries L16221 | AAATAAGACATCTCGATGGAC | Ovis aries H16386 | GCTTACCATTATGAGGATGC | 186 | 52 |
| Ovis aries L16378 | AATTGTAGCTGGACTTAACTGC | Ovis aries H16499 | GGAAGCGTGTTAAAAATGG | 166 | 54 |
| Ovis aries L16410 | TCATAATGGTAAGCATGGG | Ovis aries H16547 | GAGTATGAATTTGAGTATTGAGG | 180 | 51 |
| Ovis aries L16513 | TTTATCACCATTTTTAACACGC | Ovis aries H16573 | AGGTGCCTATATATTTACTTCGG | 104 | 54 |
| Ovis aries L16570 | CCTCAATACTCAAATTCATACTCC | Ovis aries H60 | GACCAAACCTATATGTTCATGG | 151 | 54 |
| Ovis aries L58 | TGCCTAGATGAGTCTACTGAC | Ovis aries H201 | TTAAGCAAGGCGTTGTGAG | 182 | 50 |
| Ovis aries L130 | TATACATGCAAGCATCCACG | Ovis aries H240 | TATGGCTTAATTTTTGTTACTGC | 152 | 54 |
| Ovis aries L221 | TCACAACGCCTTGCTTAACC | Ovis aries H341 | TTTACGCCGTACTCCTGTTAGC | 161 | 58 |
| Ovis aries L310 | AGGGTTGGTAAATCTCGTG | Ovis aries H407 | GTTATAATTATGGCTTTTTACAG | 138 | 50 |
| Ovis aries L374 | GCGTAAAGCGTGTTAAAGC | Ovis aries H464 | TGGGTCTTAGCTATGGTGTATC | 130 | 54 |
| Ovis aries L429 | CTGTAAAAAGCCATAATTATAAC | Ovis aries H510 | TTATTTGTGTTTAGGGCTAAG | 125 | 50 |
| Ovis aries L 484 | CTGATACACCATAGCTAAGACCC | Ovis aries H592 | AGAAGGGTATAAAGCACCGC | 149 | 54 |
| Ovis aries L14102 | GTCATCATCATTCTCACATGGAATC | Ovis aries H14223 | TGATGAAATATTTGATGGAGCTG | 168 | 55 |
| Ovis aries L14216 | ATTGTAAACAACGCATTCATTG | Ovis aries H14312 | TGTGTCAGGTGTATAGTGTATTGC | 142 | 52 |
| Ovis aries L14309 | AGATTCTAACAGGCCTATTCC | Ovis aries H14388 | GCGTGTATATATCGGATAATTC | 122 | 50 |
| Ovis aries L14362 | CATTCTCCTCTGTAACCCAC | Ovis aries H14489 | TACTCCGATGTTTCATGTTTC | 168 | 53 |
| Ovis aries L14453 | ATCTGCCTATTTATGCATGTAG | Ovis aries H14567 | TGCTCCTCAGAATGATATTTG | 157 | 53 |
| Ovis aries L14547 | AATAGCCACAGCATTCATAGG | Ovis aries H14658 | TAGCTTTGTCTACTGAGAATC | 153 | 50 |
| Ovis aries L14619 | AACCTCCTTTCAGCAATTCC | Ovis aries H14736 | GAGGAAGAGTAGGTGAACTATGG | 160 | 53 |
| Ovis aries L14729 | TTTCCCATTCATCATCGCAG | Ovis aries H14857 | GCATGAGGATGAGGATTAGTAGG | 170 | 57 |
| Ovis aries L14842 | CCACCCTTATTACACCATTAAAGAC | Ovis aries H14942 | GATGTGAGGGGGAGTGTTAAG | 146 | 55 |
| Ovis aries L14934 | CAGACAACTACACCCCAGC | Ovis aries H15058 | GGAGGAGGGGTATAATTACTAGG | 166 | 51 |
| Ovis aries L15052 | TCGCCCTAATCCTCTCAATC | Ovis aries H15159 | GCCTCCAATTCATGTGAGTG | 147 | 55 |
| Ovis aries L15147 | TGTATATTCTGAATCCTAGTAGC | Ovis aries H15218 | TGATAAGGAAATATATAATAGATGC | 119 | 50 |
| Ovis aries L15212 | ACCCCTACATCATTATTGGAC | Ovis aries H15328 | CTCCTTCTCTGGTTTACAAGAC | 159 | 53 |
