# Supplementary material for: Phylogenetic Position of a Copper Age Sheep (Ovis aries) Mitochondrial DNA
Source: PLoS One. 2012 Mar 23;7(3):e33792. doi: 10.1371/journal.pone.0033792 (PMC3311544; doi:10.1371/journal.pone.0033792)
Supplement: Table S2 — Nucleotide misincorporation rate (m) within each Copper Age sheep mtDNA group of reads. (DOC) [file pone.0033792.s005.doc]

**Table S2**. Nucleotide misincorporation rate (m) within each Copper Age sheep mtDNA group of reads.

| **Copper Age Sheep mtDNA fragment** | ***m*** |
| --- | --- |
| Ovis aries L16004/Ovis aries H16134 | 2.85×10^-3^ |
| Ovis aries L16154/Ovis aries H16267 | 1.96×10^-3^ |
| Ovis aries L16119/Ovis aries H16182 | 7.77×10^-3^ |
| Ovis aries L16221/Ovis aries L16386 | 3.36×10^-3^ |
| Ovis aries L16378/Ovis aries H16499 | 8.38×10^-3^ |
| Ovis aries L16410/Ovis aries H16547 | 9.87×10^-3^ |
| Ovis aries L16513/Ovis aries H16573 | 8.7×10^-3^ |
| Ovis aries L58/Ovis aries H201 | 3.34×10^-3^ |
| Ovis aries L130/Ovis aries H240 | 6.02×10^-3^ |
| Ovis aries L221/Ovis aries H341 | 6.35×10^-3^ |
| Ovis aries L310/Ovis aries H407 | 2.35×10^-3^ |
| Ovis aries L374/Ovis aries H464 | 2.44×10^-3^ |
| Ovis aries. L429/Ovis aries H510 | 7.82×10^-3^ |
| Ovis aries L 484/Ovis aries H592 | 7.22×10^-3^ |
| Ovis aries L14102/Ovis aries H14223 | 2.11×10^-3^ |
| Ovis aries L14216/Ovis aries H14312 | 3.36×10^-3^ |
| Ovis aries L14309/Ovis aries H14388 | 9.76×10^-3^ |
| Ovis aries L14362/Ovis aries H14489 | 3.64×10^-3^ |
| Ovis aries L14453/Ovis aries H14567 | 6.67×10^-3^ |
| Ovis aries L14547/Ovis aries H14658 | 5.62×10^-3^ |
| Ovis aries L14619/Ovis aries H14736 | 8.13×10^-3^ |
| Ovis aries L14729/Ovis aries H14857 | 4.97×10^-3^ |
| Ovis aries L14842/Ovis aries H14942 | 3.62×10^-3^ |
| Ovis aries L14934/Ovis aries H15058 | 2.48×10^-3^ |
| Ovis aries L15052/Ovis aries H15159 | 4.96×10^-3^ |
| Ovis aries L15147/Ovis aries H15218 | 1.05×10^-3^ |
| Ovis aries L15212/Ovis aries H15328 | 3.58×10^-3^ |
