# Supplementary material for: Phylogenetic Position of a Copper Age Sheep (Ovis aries) Mitochondrial DNA
Source: PLoS One. 2012 Mar 23;7(3):e33792. doi: 10.1371/journal.pone.0033792 (PMC3311544; doi:10.1371/journal.pone.0033792)
Supplement: Table S3 — Two Sample T-Tests and non-parametric Mann- Whitney tests between the values of m (nucleotide misincorporation rate) in Copper Age mtDNA, Ötzi mtDNA and modern human mtDNA. (DOC) [file pone.0033792.s006.doc]

**Table S3: Two Sample T-Tests and non-parametric Mann- Whitney tests between the values of *m*** (nucleotide misincorporation rate) in Copper Age mtDNA, Ötzi mtDNA and modern human mtDNA.

|  | **Ötzi mtDNA** | | **Modern human mtDNA** | |
| --- | --- | --- | --- | --- |
|  | Two-Sample T-Test | Mann-Whitney Test | Two-Sample T-Test | Mann-Whitney Test |
| **Copper Age sheep mtDNA** | T-value=-2.94; P-Value=0.005 | W=1808; P-Value=0.0081 | T-value=5.93; P-Value=0.000 | W=1434; P-Value=0.0000 |
