# Supplementary material for: Phylogenetic Position of a Copper Age Sheep (Ovis aries) Mitochondrial DNA
Source: PLoS One. 2012 Mar 23;7(3):e33792. doi: 10.1371/journal.pone.0033792 (PMC3311544; doi:10.1371/journal.pone.0033792)
Supplement: Table S4 — Two Sample T-Tests and non-parametric Mann- Whitney tests between the values of type 1 (A) and type 2 (B) in Copper Age sheep mtDNA, Ötzi mtDNA and modern human mtDNA. (DOC) [file pone.0033792.s007.doc]

**Table S4: Two Sample T-Tests and non-parametric Mann- Whitney tests between the values of type 1 (A) and type 2 (B) in Copper Age sheep mtDNA, Ötzi mtDNA and modern human mtDNA.**

**(A) Type 1**

|  | **Ötzi mtDNA** | | **Modern human mtDNA** | |
| --- | --- | --- | --- | --- |
|  | Two-Sample T-Test | Mann-Whitney Test | Two-Sample T-Test | Mann-Whitney Test |
| **Copper Age sheep mtDNA** | T-value=0.22; P-Value=0.826 | W=1621; P-Value=0.6654 | T-value=3.33; P-Value=0.003 | W=1866; P-Value=0.0009 |

(B) Type 2

|  | **Ötzi mtDNA** | | **Modern human mtDNA** | |
| --- | --- | --- | --- | --- |
|  | Two-Sample T-Test | Mann-Whitney Test | Two-Sample T-Test | Mann-Whitney Test |
| **Copper Age sheep mtDNA** | T-value=-2.55; P-Value=0.014 | W=737; P-Value=0.0055 | T-value=-6.30; P-Value=0.000 | W=1058; P-Value=0.0000 |
