# Supplementary material for: Phylogenetic Position of a Copper Age Sheep (Ovis aries) Mitochondrial DNA
Source: PLoS One. 2012 Mar 23;7(3):e33792. doi: 10.1371/journal.pone.0033792 (PMC3311544; doi:10.1371/journal.pone.0033792)
Supplement: Table S5 — Sequences employed to infer the Ovis aries mtDNA phylogeny including the Copper Age sheep. (DOC) [file pone.0033792.s008.doc]

**Table S5. Sequences employed to infer the *Ovis aries* mtDNA phylogeny including the copper age sheep.**

| **mtCR** | **Cytb** | **Isolation** | **Haplotype** | **Country** | **Haplogroup** | **Continent** | **Country** | **Breed** |
| --- | --- | --- | --- | --- | --- | --- | --- | --- |
| NC_001941 | NC_001941 | NC_001941 | Hap_001 | Germany | B | Europe | Germany | Merinolandschaf |
| HM236187 | (HM236187 | *Ovis vignei* isolateh76 | Hap_002 | Asia | Outgroup | Western central Asia | Asia | Urial |
| HM236174 | HM236174 | isolatecl122 | Hap_003 | Germany | A | Europe | Germany | Merino |
| HM236175 | HM236175 | isolateR359 | Hap_003 | England | A | Europe | England | Romney |
| AY879442 | AY879563 | isolateFS62 | Hap_003 | Austria | A | Europe | Austria | Forest sheep |
| AY879443 | AY879564 | isolateFS68 | Hap_003 | Austria | A | Europe | Austria | Forest sheep |
| AY879444 | AY879565 | isolateJTT89171 | Hap_003 | Indonesia | A | Asia | Indonesia | Javanese Thin Tail |
| AY879445 | AY879566 | isolateJTT90034 | Hap_003 | Indonesia | A | Asia | Indonesia | Javanese Thin Tail |
| AY879446 | AY879567 | isolateJTT90079 | Hap_003 | Indonesia | A | Asia | Indonesia | Javanese Thin Tail |
| AY879448 | AY879569 | isolateL125 | Hap_003 | Finland | A | Europe | Finland | Vepsia |
| AY879451 | AY879572 | isolateTMS14 | Hap_003 | Austria | A | Europe | Austria | Tyrolean Mountain |
| AY879453 | AY879574 | isolateTMS3 | Hap_003 | Austria | A | Europe | Austria | Tyrolean Mountain |
| AY879455 | AY879576 | isolateTMS8 | Hap_003 | Austria | A | Europe | Austria | Tyrolean Mountain |
| AY879456 | AY879577 | isolateTMS9 | Hap_003 | Austria | A | Europe | Austria | Tyrolean Mountain |
| AY879462 | AY879583 | isolateZD19 | Hap_003 | Mongolia | A | Asia | Mongolia | Tibetan |
| DQ852083 | DQ851886 | isolateKK6 | Hap_003 | Turkey | A | Middle East | Turkey | Karakas |
| DQ852093 | DQ851896 | isolatetj9 | Hap_003 | Turkey | A | Middle East | Turkey | Tuj |
| DQ852098 | DQ851901 | isolatenz1 | Hap_003 | Turkey | A | Middle East | Turkey | Norduz |
| DQ852101 | DQ851904 | isolatenz9 | Hap_003 | Turkey | A | Middle East | Turkey | Norduz |
| DQ852104 | DQ851907 | isolatenz20 | Hap_003 | Turkey | A | Middle East | Turkey | Norduz |
| DQ852114 | DQ851917 | isolateAW6 | Hap_003 | Israel | A | Middle East | Israel | Awassi |
| DQ852115 | DQ851918 | isolateAW8 | Hap_003 | Israel | A | Middle East | Israel | Awassi |
| DQ852117 | DQ851920 | isolateAW26 | Hap_003 | Israel | A | Middle East | Israel | Awassi |
| DQ852127 | DQ851930 | isolateAW60 | Hap_003 | Israel | A | Middle East | Israel | Awassi |
| EF056431 | FJ218058 | isolateGa_G9 | Hap_003 | India | A | Asia | India | Garole |
| HM236176 | HM236176 | isolatekk1 | Hap_004 | Turkey | B | Middle East | Turkey | Karakas |
| HM236177 | HM236177 | isolatekk2 | Hap_004 | Turkey | B | Middle East | Turkey | Karakas |
| AY879383 | AY879504 | isolateJTT91113 | Hap_004 | Indonesia | B | Asia | Indonesia | Javanese Thin Tail |
| AY879393 | AY879514 | isolateL112 | Hap_004 | Finland | B | Europe | Finland | Vepsia |
| AY879394 | AY879515 | isolateL127 | Hap_004 | Finland | B | Europe | Finland | Vepsia |
| AY879415 | AY879536 | isolateTMS19 | Hap_004 | Austria | B | Europe | Austria | Tyrolean Mountain |
| AY879428 | AY879549 | isolateTSS31 | Hap_004 | Austria | B | Europe | Austria | Tyrolean Stone |
| DQ852130 | DQ851933 | isolatekk4 | Hap_004 | Turkey | B | Middle East | Turkey | Karakas |
| DQ852131 | DQ851934 | isolatekk5 | Hap_004 | Turkey | B | Middle East | Turkey | Karakas |
| DQ852135 | DQ851938 | isolatekk11 | Hap_004 | Turkey | B | Middle East | Turkey | Karakas |
| DQ852139 | DQ851942 | isolatekk20 | Hap_004 | Turkey | B | Middle East | Turkey | Karakas |
| DQ852148 | DQ851951 | isolatemk16 | Hap_004 | Turkey | B | Middle East | Turkey | Morkaraman |
| DQ852159 | DQ851962 | isolatekr2 | Hap_004 | Turkey | B | Middle East | Turkey | Karya |
| DQ852161 | DQ851964 | isolatekr4 | Hap_004 | Turkey | B | Middle East | Turkey | Karya |
| DQ852171 | DQ851974 | isolatekr15 | Hap_004 | Turkey | B | Middle East | Turkey | Karya |
| DQ852175 | DQ851978 | isolatekr19 | Hap_004 | Turkey | B | Middle East | Turkey | Karya |
| DQ852180 | DQ851983 | isolatenz7 | Hap_004 | Turkey | B | Middle East | Turkey | Norduz |
| DQ852181 | DQ851984 | isolatenz16 | Hap_004 | Turkey | B | Middle East | Turkey | Norduz |
| DQ852182 | DQ851985 | isolatenz19 | Hap_004 | Turkey | B | Middle East | Turkey | Norduz |
| DQ852186 | DQ851989 | isolatecc64 | Hap_004 | Turkey | B | Middle East | Turkey | Cine Capari |
| DQ852188 | DQ851991 | isolatecc300 | Hap_004 | Turkey | B | Middle East | Turkey | Cine Capari |
| DQ852192 | DQ851995 | isolateky5 | Hap_004 | Turkey | B | Middle East | Turkey | Karayaka |
| DQ852196 | DQ851999 | isolateky12 | Hap_004 | Turkey | B | Middle East | Turkey | Karayaka |
| DQ852216 | DQ852019 | isolateAW1 | Hap_004 | Israel | B | Middle East | Israel | Awassi |
| DQ852217 | DQ852020 | isolateAW5 | Hap_004 | Israel | B | Middle East | Israel | Awassi |
| DQ852219 | DQ852022 | isolateAW9 | Hap_004 | Israel | B | Middle East | Israel | Awassi |
| DQ852232 | DQ852035 | isolateAW32 | Hap_004 | Israel | B | Middle East | Israel | Awassi |
| DQ852237 | DQ852040 | isolateAW42 | Hap_004 | Israel | B | Middle East | Israel | Awassi |
| DQ852242 | DQ852045 | isolateAW50 | Hap_004 | Israel | B | Middle East | Israel | Awassi |
| DQ852246 | DQ852049 | isolateAW63 | Hap_004 | Israel | B | Middle East | Israel | Awassi |
| HM236178 | HM236178 | isolatekk12 | Hap_005 | Turkey | C | Middle East | Turkey | Karakas |
| HM236179 | HM236179 | isolatemk4 | Hap_005 | Turkey | C | Middle East | Turkey | Morkaraman |
| DQ852249 | DQ852052 | isolatekk13 | Hap_005 | Turkey | C | Middle East | Turkey | Karakas |
| DQ852250 | DQ852053 | isolatekk19 | Hap_005 | Turkey | C | Middle East | Turkey | Karakas |
| DQ852261 | DQ852064 | isolatecc21 | Hap_005 | Turkey | C | Middle East | Turkey | Cine Capari |
| DQ852262 | DQ852065 | isolatecc51 | Hap_005 | Turkey | C | Middle East | Turkey | Cine Capari |
| DQ852266 | DQ852069 | isolateAW3 | Hap_005 | Israel | C | Middle East | Israel | Awassi |
| DQ852267 | DQ852070 | isolateAW4 | Hap_005 | Israel | C | Middle East | Israel | Awassi |
| DQ852268 | DQ852071 | isolateAW30 | Hap_005 | Israel | C | Middle East | Israel | Awassi |
| DQ852270 | DQ852073 | isolateAW41 | Hap_005 | Israel | C | Middle East | Israel | Awassi |
| DQ852271 | DQ852074 | isolateAW51 | Hap_005 | Israel | C | Middle East | Israel | Awassi |
| DQ852272 | DQ852075 | isolateAW58 | Hap_005 | Israel | C | Middle East | Israel | Awassi |
| HM236180 | HM236180 | isolatemk3 | Hap_006 | Turkey | D* | Middle East | Turkey | Morkaraman |
| HM236181 | HM236181 | isolatemk9 | Hap_006 | Turkey | D* | Middle East | Turkey | Morkaraman |
| HM236182 | HM236182 | isolateaw25 | Hap_007 | Israel | E | Middle East | Israel | Awassi |
| DQ852277 | DQ852078 | isolateAW12 | Hap_007 | Israel | E | Middle East | Israel | Awassi |
| DQ852278 | DQ852079 | isolateAW24 | Hap_007 | Israel | E | Middle East | Israel | Awassi |
| HM236183 | HM236183 | isolatetj6 | Hap_008 | Turkey | E | Middle East | Turkey | Tuj |
| Iceman's_sheep | Iceman's_sheep | Iceman's_sheep | Hap_009 | Italy | B | Europe | Italy | 0 |
| AY879343 | AY879464 | isolateCS52 | Hap_010 | Austria | B | Europe | Austria | Carynthian sheep |
| AY879344 | AY879465 | isolateAH11 | Hap_011 | Åland Island | B | Europe | Åland Island | Aland |
| AY879345 | AY879466 | isolateAH18 | Hap_011 | Åland Island | B | Europe | Åland Island | Aland |
| AY879346 | AY879467 | isolateAH8 | Hap_012 | Åland Island | B | Europe | Åland Island | Aland |
| AY879396 | AY879517 | isolateL199 | Hap_012 | Åland Island | B | Europe | Åland Island | Aland |
| AY879347 | AY879468 | isolateCS41 | Hap_013 | Austria | B | Europe | Austria | Carynthian sheep |
| AY879357 | AY879478 | isolateCS53 | Hap_013 | Austria | B | Europe | Austria | Carynthian sheep |
| AY879358 | AY879479 | isolateCS54 | Hap_013 | Austria | B | Europe | Austria | Carynthian sheep |
| AY879359 | AY879480 | isolateCS56 | Hap_013 | Austria | B | Europe | Austria | Carynthian sheep |
| AY879348 | AY879469 | isolateCS42 | Hap_014 | Austria | B | Europe | Austria | Carynthian sheep |
| AY879349 | AY879470 | isolateCS43 | Hap_015 | Austria | B | Europe | Austria | Carynthian sheep |
| AY879350 | AY879471 | isolateCS44 | Hap_016 | Austria | B | Europe | Austria | Carynthian sheep |
| AY879351 | AY879472 | isolateCS46 | Hap_016 | Austria | B | Europe | Austria | Carynthian sheep |
| AY879352 | AY879473 | isolateCS47 | Hap_016 | Austria | B | Europe | Austria | Carynthian sheep |
| AY879353 | AY879474 | isolateCS48 | Hap_016 | Austria | B | Europe | Austria | Carynthian sheep |
| AY879354 | AY879475 | isolateCS49 | Hap_016 | Austria | B | Europe | Austria | Carynthian sheep |
| AY879355 | AY879476 | isolateCS50 | Hap_016 | Austria | B | Europe | Austria | Carynthian sheep |
| AY879356 | AY879477 | isolateCS51 | Hap_016 | Austria | B | Europe | Austria | Carynthian sheep |
| AY879361 | AY879482 | isolateCS58 | Hap_016 | Austria | B | Europe | Austria | Carynthian sheep |
| AY879362 | AY879483 | isolateCS59 | Hap_016 | Austria | B | Europe | Austria | Carynthian sheep |
| AY879363 | AY879484 | isolateCS60 | Hap_016 | Austria | B | Europe | Austria | Carynthian sheep |
| AY879360 | AY879481 | isolateCS57 | Hap_017 | Austria | B | Europe | Austria | Carynthian sheep |
| AY879364 | AY879485 | isolateFS61 | Hap_018 | Austria | B | Europe | Austria | Forest sheep |
| AY879367 | AY879488 | isolateFS66 | Hap_018 | Austria | B | Europe | Austria | Forest sheep |
| AY879369 | AY879490 | isolateFS69 | Hap_018 | Austria | B | Europe | Austria | Forest sheep |
| AY879370 | AY879491 | isolateFS | Hap_018 | Austria | B | Europe | Austria | Forest sheep |
| AY879371 | AY879492 | isolateFS71 | Hap_018 | Austria | B | Europe | Austria | Forest sheep |
| AY879365 | AY879486 | isolateFS63 | Hap_019 | Austria | B | Europe | Austria | Forest sheep |
| AY879366 | AY879487 | isolateFS64 | Hap_020 | Austria | B | Europe | Austria | Forest sheep |
| AY879372 | AY879493 | isolateFS72 | Hap_020 | Austria | B | Europe | Austria | Forest sheep |
| AY879368 | AY879489 | isolateFS67 | Hap_021 | Austria | B | Europe | Austria | Forest sheep |
| AY879373 | AY879494 | isolateFS73 | Hap_022 | Austria | B | Europe | Austria | Forest sheep |
| AY879374 | AY879495 | isolateJTT87346 | Hap_023 | Indonesia | B | Asia | Indonesia | Javanese Thin Tail |
| AY879375 | AY879496 | isolateJTT89205 | Hap_023 | Indonesia | B | Asia | Indonesia | Javanese Thin Tail |
| AY879376 | AY879497 | isolateJTT89344 | Hap_023 | Indonesia | B | Asia | Indonesia | Javanese Thin Tail |
| AY879377 | AY879498 | isolateJTT89424 | Hap_023 | Indonesia | B | Asia | Indonesia | Javanese Thin Tail |
| AY879378 | AY879499 | isolateJTT90057 | Hap_023 | Indonesia | B | Asia | Indonesia | Javanese Thin Tail |
| AY879379 | AY879500 | isolateJTT90125 | Hap_023 | Indonesia | B | Asia | Indonesia | Javanese Thin Tail |
| AY879381 | AY879502 | isolateJTT91020 | Hap_023 | Indonesia | B | Asia | Indonesia | Javanese Thin Tail |
| AY879382 | AY879503 | isolateJTT91021 | Hap_023 | Indonesia | B | Asia | Indonesia | Javanese Thin Tail |
| AY879385 | AY879506 | isolateJTT92068 | Hap_023 | Indonesia | B | Asia | Indonesia | Javanese Thin Tail |
| AY879386 | AY879507 | isolateJTT92123 | Hap_023 | Indonesia | B | Asia | Indonesia | Javanese Thin Tail |
| AY879387 | AY879508 | isolateJTT92127 | Hap_023 | Indonesia | B | Asia | Indonesia | Javanese Thin Tail |
| AY879380 | AY879501 | isolateJTT91014 | Hap_024 | Indonesia | B | Asia | Indonesia | Javanese Thin Tail |
| AY879384 | AY879505 | isolateJTT92041 | Hap_025 | Indonesia | B | Asia | Indonesia | Javanese Thin Tail |
| AY879388 | AY879509 | isolateL101 | Hap_026 | Finland | B | Europe | Finland | Viena |
| AY879389 | AY879510 | isolatel107 | Hap_026 | Finland | B | Europe | Finland | Viena |
| AY879390 | AY879511 | isolateL108 | Hap_026 | Finland | B | Europe | Finland | Viena |
| AY879391 | AY879512 | isolateL109 | Hap_026 | Finland | B | Europe | Finland | Viena |
| AY879398 | AY879519 | isolateL33 | Hap_026 | Finland | B | Europe | Finland | Viena |
| AY879392 | AY879513 | isolateL110 | Hap_027 | Finland | B | Europe | Finland | Vepsia |
| AY879395 | AY879516 | isolateL128 | Hap_028 | Finland | B | Europe | Finland | Vepsia |
| AY879397 | AY879518 | isolate2691 | Hap_029 | Australia | B | Oceania | Australia | Suffolk |
| AY879399 | AY879520 | isolateL35 | Hap_030 | Finland | B | Europe | Finland | Grey Finnsheep |
| AY879400 | AY879521 | isolateL449 | Hap_031 | Australia | B | Oceania | Australia | Suffolk |
| AY879401 | AY879522 | isolateL44 | Hap_032 | Finland | B | Europe | Finland | White Finnsheep |
| AY879402 | AY879523 | isolateL93 | Hap_033 | Finland | B | Europe | Finland | Brown Finnsheep |
| AY879403 | AY879524 | isolateL95 | Hap_034 | Finland | B | Europe | Finland | Black Finnsheep |
| AY879404 | AY879525 | isolateL98 | Hap_035 | Finland | B | Europe | Finland | Viena |
| AY879405 | AY879526 | isolateLAF2 | Hap_036 | Finland | B | Europe | Finland | White Finnsheep |
| AY879406 | AY879527 | isolateLAK1 | Hap_037 | Australia | B | Oceania | Australia | Karakul |
| AY879407 | AY879528 | isolate015 | Hap_038 | Finland | B | Europe | Finland | Oxford Down |
| AY879408 | AY879529 | isolateO1 | Hap_039 | Finland | B | Europe | Finland | Oxford Down |
| AY879413 | AY879534 | isolateTMS15 | Hap_040 | Austria | B | Europe | Austria | Tyrolean Mountain |
| AY879418 | AY879539 | isolateTMS4 | Hap_040 | Austria | B | Europe | Austria | Tyrolean Mountain |
| AY879414 | AY879535 | isolateTMS17 | Hap_041 | Austria | B | Europe | Austria | Tyrolean Mountain |
| AY879417 | AY879538 | isolateTMS20 | Hap_042 | Austria | B | Europe | Austria | Tyrolean Mountain |
| AY879420 | AY879541 | isolateTMS6 | Hap_043 | Austria | B | Europe | Austria | Tyrolean Mountain |
| AY879421 | AY879542 | isolateTSS21 | Hap_044 | Austria | B | Europe | Austria | Tyrolean Stone |
| DQ852165 | DQ851968 | isolatekr8 | Hap_044 | Turkey | B | Middle East | Turkey | Karya |
| DQ852167 | DQ851970 | isolatekr10 | Hap_044 | Turkey | B | Middle East | Turkey | Karya |
| DQ852173 | DQ851976 | isolatekr17 | Hap_044 | Turkey | B | Middle East | Turkey | Karya |
| DQ852174 | DQ851977 | isolatekr18 | Hap_044 | Turkey | B | Middle East | Turkey | Karya |
| DQ852177 | DQ851980 | isolatekr22 | Hap_044 | Turkey | B | Middle East | Turkey | Karya |
| AY879422 | AY879543 | isolateTSS22 | Hap_045 | Austria | B | Europe | Austria | Tyrolean Stone |
| AY879423 | AY879544 | isolateTSS23 | Hap_045 | Austria | B | Europe | Austria | Tyrolean Stone |
| AY879431 | AY879552 | isolateTSS38 | Hap_045 | Austria | B | Europe | Austria | Tyrolean Stone |
| AY879424 | AY879545 | isolateTSS24 | Hap_046 | Austria | B* | Europe | Austria | Tyrolean Stone |
| AY879425 | AY879546 | isolateTSS27 | Hap_046 | Austria | B* | Europe | Austria | Tyrolean Stone |
| AY879427 | AY879548 | isolateTSS29 | Hap_046 | Austria | B* | Europe | Austria | Tyrolean Stone |
| AY879432 | AY879553 | isolateTSS40 | Hap_046 | Austria | B* | Europe | Austria | Tyrolean Stone |
| AY879426 | AY879547 | isolateTSS28 | Hap_047 | Austria | B | Europe | Austria | Tyrolean Stone |
| AY879429 | AY879550 | isolateTSS32 | Hap_048 | Austria | B | Europe | Austria | Tyrolean Stone |
| AY879430 | AY879551 | isolateTSS37 | Hap_049 | Austria | B* | Europe | Austria | Tyrolean Stone |
| AY879433 | AY879554 | isolateZB13 | Hap_050 | Mongolia | B | Asia | Mongolia | Tibetan |
| AY879434 | AY879555 | isolateLX106 | Hap_051 | Spain | B | Europe | Spain | Latxa |
| AY879435 | AY879556 | isolateLX187 | Hap_052 | Spain | B | Europe | Spain | Latxa |
| AY879440 | AY879561 | isolateAH25 | Hap_053 | Åland Island | A | Europe | Åland Island | Aland |
| AY879447 | AY879568 | isolateJTT92090 | Hap_054 | Indonesia | A | Asia | Indonesia | Javanese Thin Tail |
| AY879449 | AY879570 | isolateL8991 | Hap_055 | Australia | A | Oceania | Australia | Suffolk |
| AY879452 | AY879573 | isolateTMS18 | Hap_056 | Austria | A | Europe | Austria | Tyrolean Mountain |
| AY879454 | AY879575 | isolateTMS7 | Hap_057 | Austria | A | Europe | Austria | Tyrolean Mountain |
| AY879457 | AY879578 | isolateTSS36 | Hap_058 | Austria | A | Europe | Austria | Tyrolean Stone |
| AY879458 | AY879579 | isolateZB09 | Hap_059 | Mongolia | A | Asia | Mongolia | Tibetan |
| AY879459 | AY879580 | isolateZD04 | Hap_060 | Mongolia | A | Asia | Mongolia | Tibetan |
| AY879460 | AY879581 | isolateZD11 | Hap_061 | Mongolia | A | Asia | Mongolia | Tibetan |
| DQ852109 | DQ851912 | isolateky9 | Hap_061 | Turkey | A | Middle East | Turkey | Karayaka |
| DQ852110 | DQ851913 | isolateky10 | Hap_061 | Turkey | A | Middle East | Turkey | Karayaka |
| DQ852112 | DQ851915 | isolateky14 | Hap_061 | Turkey | A | Middle East | Turkey | Karayaka |
| AY879461 | AY879582 | isolateZD15 | Hap_062 | Mongolia | A | Asia | Mongolia | Tibetan |
| AY879463 | AY879584 | isolatemongolian1 | Hap_063 | Mongolia | A | Asia | Mongolia | Mongolian |
| DQ852222 | DQ852025 | isolateAW13 | Hap_064 | Israel | B | Middle East | Israel | Awassi |
| DQ852222 | DQ852025 | isolateAW13 | Hap_064 | Israel | B | Middle East | Israel | Awassi |
| DQ852233 | DQ852036 | isolateAW37 | Hap_064 | Israel | B | Middle East | Israel | Awassi |
| DQ852223 | DQ852026 | isolateAW15 | Hap_065 | Israel | B | Middle East | Israel | Awassi |
| DQ852220 | DQ852023 | isolateAW10 | Hap_065 | Israel | B | Middle East | Israel | Awassi |
| DQ852223 | DQ852026 | isolateAW15 | Hap_065 | Israel | B | Middle East | Israel | Awassi |
| DQ852087 | DQ851890 | isolateMK5 | Hap_066 | Turkey | A | Middle East | Turkey | Morkaraman |
| DQ852090 | DQ851893 | isolatemk14 | Hap_066 | Turkey | A | Middle East | Turkey | Morkaraman |
| EF056417 | FJ218039 | isolateGa_16 | Hap_066 | India | A | Asia | India | Garole |
| DQ852088 | DQ851891 | isolatemk11 | Hap_067 | Turkey | A | Middle East | Turkey | Morkaraman |
| DQ852089 | DQ851892 | isolatemk13 | Hap_067 | Turkey | A | Middle East | Turkey | Morkaraman |
| DQ852091 | DQ851894 | isolatemk19 | Hap_067 | Turkey | A | Middle East | Turkey | Morkaraman |
| DQ852092 | DQ851895 | isolatetj5 | Hap_067 | Turkey | A | Middle East | Turkey | Tuj |
| DQ852094 | DQ851897 | isolatetj13 | Hap_068 | Turkey | A | Middle East | Turkey | Tuj |
| DQ852095 | DQ851898 | isolatekr13 | Hap_069 | Turkey | A | Middle East | Turkey | Karya |
| DQ852096 | DQ851899 | isolatekr20 | Hap_069 | Turkey | A | Middle East | Turkey | Karya |
| DQ852097 | DQ851900 | isolatekr24 | Hap_069 | Turkey | A | Middle East | Turkey | Karya |
| DQ852107 | DQ851910 | isolateky1 | Hap_069 | Turkey | A | Middle East | Turkey | Karayaka |
| DQ852099 | DQ851902 | isolatenz2 | Hap_070 | Turkey | A | Middle East | Turkey | Norduz |
| DQ852100 | DQ851903 | isolatenz4 | Hap_070 | Turkey | A | Middle East | Turkey | Norduz |
| DQ852105 | DQ851908 | isolatecc53 | Hap_071 | Turkey | A | Middle East | Turkey | Cine Capari |
| DQ852106 | DQ851909 | isolatecc304 | Hap_072 | Turkey | A | Middle East | Turkey | Cine Capari |
| DQ852108 | DQ851911 | isolateky4 | Hap_073 | Turkey | A | Middle East | Turkey | Karayaka |
| DQ852111 | DQ851914 | isolateky11 | Hap_074 | Turkey | A | Middle East | Turkey | Karayaka |
| DQ852113 | DQ851916 | isolateAW2 | Hap_075 | Israel | A | Middle East | Israel | Awassi |
| DQ852116 | DQ851919 | isolateAW21 | Hap_075 | Israel | A | Middle East | Israel | Awassi |
| DQ852122 | DQ851925 | isolateAW43 | Hap_075 | Israel | A | Middle East | Israel | Awassi |
| DQ852126 | DQ851929 | isolateAW59 | Hap_075 | Israel | A | Middle East | Israel | Awassi |
| DQ852118 | DQ851921 | isolateAW27 | Hap_076 | Israel | A | Middle East | Israel | Awassi |
| DQ852119 | DQ851922 | isolateAW33 | Hap_077 | Israel | A | Middle East | Israel | Awassi |
| DQ852120 | DQ851923 | isolateAW35 | Hap_078 | Israel | A | Middle East | Israel | Awassi |
| DQ852123 | DQ851926 | isolateAW44 | Hap_079 | Israel | A | Middle East | Israel | Awassi |
| DQ852132 | DQ851935 | isolatekk8 | Hap_080 | Turkey | B | Middle East | Turkey | Karakas |
| DQ852133 | DQ851936 | isolatekk9 | Hap_080 | Turkey | B | Middle East | Turkey | Karakas |
| DQ852136 | DQ851939 | isolatekk14 | Hap_080 | Turkey | B | Middle East | Turkey | Karakas |
| DQ852136 | DQ851939 | isolatekk14 | Hap_080 | Turkey | B | Middle East | Turkey | Karakas |
| DQ852138 | DQ851941 | isolatekk17 | Hap_080 | Turkey | B | Middle East | Turkey | Karakas |
| DQ852134 | DQ851937 | isolatekk10 | Hap_081 | Turkey | B | Middle East | Turkey | Karakas |
| DQ852137 | DQ851940 | isolatekk15 | Hap_082 | Turkey | B | Middle East | Turkey | Karakas |
| DQ852140 | DQ851943 | isolatemk1 | Hap_083 | Turkey | B | Middle East | Turkey | Morkaraman |
| DQ852143 | DQ851946 | isolatemk7 | Hap_083 | Turkey | B | Middle East | Turkey | Morkaraman |
| DQ852146 | DQ851949 | isolatemk12 | Hap_083 | Turkey | B | Middle East | Turkey | Morkaraman |
| DQ852147 | DQ851950 | isolatemk15 | Hap_083 | Turkey | B | Middle East | Turkey | Morkaraman |
| DQ852149 | DQ851952 | isolatemk17 | Hap_083 | Turkey | B | Middle East | Turkey | Morkaraman |
| DQ852142 | DQ851945 | isolatemk6 | Hap_084 | Turkey | B | Middle East | Turkey | Morkaraman |
| DQ852144 | DQ851947 | isolatemk8 | Hap_085 | Turkey | B | Middle East | Turkey | Morkaraman |
| DQ852145 | DQ851948 | isolatemk10 | Hap_086 | Turkey | B | Middle East | Turkey | Morkaraman |
| DQ852150 | DQ851953 | isolatemk20 | Hap_086 | Turkey | B | Middle East | Turkey | Morkaraman |
| DQ852151 | DQ851954 | isolatetj3 | Hap_087 | Turkey | B | Middle East | Turkey | Tuj |
| DQ852152 | DQ851955 | isolatetj4 | Hap_088 | Turkey | B | Middle East | Turkey | Tuj |
| DQ852153 | DQ851956 | isolatetj7 | Hap_088 | Turkey | B | Middle East | Turkey | Tuj |
| DQ852154 | DQ851957 | isolatetj8 | Hap_089 | Turkey | B | Middle East | Turkey | Tuj |
| DQ852155 | DQ851958 | isolatetj11 | Hap_090 | Turkey | B | Middle East | Turkey | Tuj |
| DQ852157 | DQ851960 | isolatetj14 | Hap_091 | Turkey | B | Middle East | Turkey | Tuj |
| DQ852158 | DQ851961 | isolatekr1 | Hap_092 | Turkey | B | Middle East | Turkey | Karya |
| DQ852190 | DQ851993 | isolateky2 | Hap_092 | Turkey | B | Middle East | Turkey | Karayaka |
| DQ852199 | DQ852002 | isolatesz4 | Hap_092 | Turkey | B | Middle East | Turkey | Sakiz |
| DQ852200 | DQ852003 | isolatesz5 | Hap_092 | Turkey | B | Middle East | Turkey | Sakiz |
| DQ852201 | DQ852004 | isolatesz6 | Hap_092 | Turkey | B | Middle East | Turkey | Sakiz |
| DQ852202 | DQ852005 | isolatesz8 | Hap_092 | Turkey | B | Middle East | Turkey | Sakiz |
| DQ852203 | DQ852006 | isolatesz9 | Hap_092 | Turkey | B | Middle East | Turkey | Sakiz |
| DQ852204 | DQ852007 | isolatesz13 | Hap_092 | Turkey | B | Middle East | Turkey | Sakiz |
| DQ852205 | DQ852008 | isolatesz16 | Hap_092 | Turkey | B | Middle East | Turkey | Sakiz |
| DQ852207 | DQ852010 | isolatesz20 | Hap_092 | Turkey | B | Middle East | Turkey | Sakiz |
| DQ852208 | DQ852011 | isolatesz21 | Hap_092 | Turkey | B | Middle East | Turkey | Sakiz |
| DQ852209 | DQ852012 | isolatesz22 | Hap_092 | Turkey | B | Middle East | Turkey | Sakiz |
| DQ852210 | DQ852013 | isolatesz24 | Hap_092 | Turkey | B | Middle East | Turkey | Sakiz |
| DQ852211 | DQ852014 | isolatesz27 | Hap_092 | Turkey | B | Middle East | Turkey | Sakiz |
| DQ852212 | DQ852015 | isolatesz31 | Hap_092 | Turkey | B | Middle East | Turkey | Sakiz |
| DQ852213 | DQ852016 | isolatesz34 | Hap_092 | Turkey | B | Middle East | Turkey | Sakiz |
| DQ852214 | DQ852017 | isolatesz35 | Hap_092 | Turkey | B | Middle East | Turkey | Sakiz |
| DQ852160 | DQ851963 | isolatekr3 | Hap_093 | Turkey | B | Middle East | Turkey | Karya |
| DQ852162 | DQ851965 | isolatekr5 | Hap_094 | Turkey | B | Middle East | Turkey | Karya |
| DQ852163 | DQ851966 | isolatekr6 | Hap_095 | Turkey | B | Middle East | Turkey | Karya |
| DQ852164 | DQ851967 | isolatekr7 | Hap_096 | Turkey | B* | Middle East | Turkey | Karya |
| DQ852166 | DQ851969 | isolatekr9 | Hap_097 | Turkey | B | Middle East | Turkey | Karya |
| DQ852168 | DQ851971 | isolatekr11 | Hap_098 | Turkey | B | Middle East | Turkey | Karya |
| DQ852169 | DQ851972 | isolatekr12 | Hap_099 | Turkey | B | Middle East | Turkey | Karya |
| DQ852170 | DQ851973 | isolatekr14 | Hap_099 | Turkey | B | Middle East | Turkey | Karya |
| DQ852178 | DQ851981 | isolatekr23 | Hap_099 | Turkey | B | Middle East | Turkey | Karya |
| DQ852172 | DQ851975 | isolatekr16 | Hap_100 | Turkey | B | Middle East | Turkey | Karya |
| DQ852176 | DQ851979 | isolatekr21 | Hap_101 | Turkey | B | Middle East | Turkey | Karya |
| DQ852185 | DQ851988 | isolatecc58 | Hap_101 | Turkey | B | Middle East | Turkey | Cine Capari |
| DQ852179 | DQ851982 | isolatenz3 | Hap_102 | Turkey | B | Middle East | Turkey | Norduz |
| DQ852183 | DQ851986 | isolatecc31 | Hap_103 | Turkey | B* | Middle East | Turkey | Cine Capari |
| DQ852184 | DQ851987 | isolatecc50 | Hap_104 | Turkey | B | Middle East | Turkey | Cine Capari |
| DQ852187 | DQ851990 | isolatecc224 | Hap_105 | Turkey | B | Middle East | Turkey | Cine Capari |
| DQ852189 | DQ851992 | isolatecc301 | Hap_106 | Turkey | B | Middle East | Turkey | Cine Capari |
| DQ852191 | DQ851994 | isolateky3 | Hap_107 | Turkey | B | Middle East | Turkey | Karayaka |
| DQ852193 | DQ851996 | isolateky6 | Hap_108 | Turkey | B | Middle East | Turkey | Karayaka |
| DQ852197 | DQ852000 | isolateky13 | Hap_108 | Turkey | B | Middle East | Turkey | Karayaka |
| DQ852194 | DQ851997 | isolateky7 | Hap_109 | Turkey | B | Middle East | Turkey | Karayaka |
| DQ852195 | DQ851998 | isolateky8 | Hap_109 | Turkey | B | Middle East | Turkey | Karayaka |
| DQ852198 | DQ852001 | isolateky16 | Hap_110 | Turkey | B | Middle East | Turkey | Karayaka |
| DQ852221 | DQ852024 | isolateAW11 | Hap_111 | Israel | B | Middle East | Israel | Awassi |
| DQ852231 | DQ852034 | isolateAW29 | Hap_111 | Israel | B | Middle East | Israel | Awassi |
| DQ852206 | DQ852009 | isolatesz17 | Hap_112 | Turkey | B | Middle East | Turkey | Sakiz |
| DQ852215 | DQ852018 | isolatesz36 | Hap_112 | Turkey | B | Middle East | Turkey | Sakiz |
| DQ852218 | DQ852021 | isolateAW7 | Hap_113 | Israel | B | Middle East | Israel | Awassi |
| DQ852235 | DQ852038 | isolateAW39 | Hap_113 | Israel | B | Middle East | Israel | Awassi |
| DQ852224 | DQ852027 | isolateAW17 | Hap_114 | Israel | B | Middle East | Israel | Awassi |
| DQ852226 | DQ852029 | isolateAW19 | Hap_115 | Israel | B | Middle East | Israel | Awassi |
| DQ852227 | DQ852030 | isolateAW20 | Hap_116 | Israel | B | Middle East | Israel | Awassi |
| DQ852239 | DQ852042 | isolateAW46 | Hap_116 | Israel | B | Middle East | Israel | Awassi |
| DQ852241 | DQ852044 | isolateAW49 | Hap_116 | Israel | B | Middle East | Israel | Awassi |
| DQ852228 | DQ852031 | isolateAW22 | Hap_117 | Israel | B | Middle East | Israel | Awassi |
| DQ852230 | DQ852033 | isolateAW28 | Hap_118 | Israel | B* | Middle East | Israel | Awassi |
| DQ852234 | DQ852037 | isolateAW38 | Hap_119 | Israel | B | Middle East | Israel | Awassi |
| DQ852236 | DQ852039 | isolateAW40 | Hap_120 | Israel | B* | Middle East | Israel | Awassi |
| DQ852240 | DQ852043 | isolateAW47 | Hap_120 | Israel | B* | Middle East | Israel | Awassi |
| DQ852244 | DQ852047 | isolateAW53 | Hap_120 | Israel | B* | Middle East | Israel | Awassi |
| DQ852245 | DQ852048 | isolateAW55 | Hap_120 | Israel | B* | Middle East | Israel | Awassi |
| DQ852238 | DQ852041 | isolateAW45 | Hap_121 | Israel | B | Middle East | Israel | Awassi |
| DQ852243 | DQ852046 | isolateAW52 | Hap_121 | Israel | B | Middle East | Israel | Awassi |
| DQ852247 | DQ852050 | isolatekk3 | Hap_122 | Turkey | C | Middle East | Turkey | Karakas |
| DQ852254 | DQ852057 | isolatetj10 | Hap_122 | Turkey | C | Middle East | Turkey | Tuj |
| DQ852256 | DQ852059 | isolatetj16 | Hap_122 | Turkey | C | Middle East | Turkey | Tuj |
| DQ852252 | DQ852055 | isolatetj1 | Hap_123 | Turkey | C | Middle East | Turkey | Tuj |
| DQ852253 | DQ852056 | isolatetj2 | Hap_123 | Turkey | C | Middle East | Turkey | Tuj |
| DQ852257 | DQ852060 | isolatenz5 | Hap_124 | Turkey | C | Middle East | Turkey | Norduz |
| DQ852258 | DQ852061 | isolatenz8 | Hap_125 | Turkey | C | Middle East | Turkey | Norduz |
| DQ852259 | DQ852062 | isolatenz13 | Hap_125 | Turkey | C | Middle East | Turkey | Norduz |
| DQ852260 | DQ852063 | isolatenz14 | Hap_126 | Turkey | C | Middle East | Turkey | Norduz |
| DQ852263 | DQ852066 | isolatecc202 | Hap_127 | Turkey | C | Middle East | Turkey | Cine Capari |
| DQ852264 | DQ852067 | isolatecc226 | Hap_127 | Turkey | C | Middle East | Turkey | Cine Capari |
| DQ852269 | DQ852072 | isolateAW31 | Hap_128 | Israel | C | Middle East | Israel | Awassi |
| DQ852273 | DQ852076 | isolateAW62 | Hap_128 | Israel | C | Middle East | Israel | Awassi |
| EF056397 | FJ218023 | isolateBn_260 | Hap_129 | India | A | Asia | India | Bannur |
| EF056400 | FJ218035 | isolateBn_34009 | Hap_130 | India | A | Asia | India | Bannur |
| EF056402 | FJ218026 | isolateBn_502 | Hap_131 | India | A | Asia | India | Bannur |
| EF056403 | FJ218027 | isolateBn_509 | Hap_132 | India | A | Asia | India | Bannur |
| EF056410 | FJ218034 | isolateBn_566 | Hap_132 | India | A | Asia | India | Bannur |
| EF056404 | FJ218028 | isolateBn_512 | Hap_133 | India | A | Asia | India | Bannur |
| EF056405 | FJ218029 | isolateBn_527 | Hap_134 | India | A | Asia | India | Bannur |
| EF056407 | FJ218032 | isolateBn_544 | Hap_135 | India | A | Asia | India | Bannur |
| EF056408 | FJ218033 | isolateBn_552 | Hap_136 | India | A | Asia | India | Bannur |
| EF056412 | FJ218036 | isolateBn_D98036 | Hap_137 | India | A | Asia | India | Bannur |
| EF056415 | FJ218043 | isolateGa_142 | Hap_138 | India | A | Asia | India | Garole |
| EF056418 | FJ218050 | isolateGa_16222 | Hap_139 | India | A | Asia | India | Garole |
| EF056419 | FJ218051 | isolateGa_16251 | Hap_140 | India | A | Asia | India | Garole |
| EF056421 | FJ218053 | isolateGa_16283 | Hap_141 | India | A | Asia | India | Garole |
| EF056423 | FJ218054 | isolateGa_16290 | Hap_142 | India | A | Asia | India | Garole |
| EF056424 | FJ218057 | isolateGa_34743 | Hap_143 | India | A | Asia | India | Garole |
| EF056425 | FJ218040 | isolateGa_40 | Hap_144 | India | A | Asia | India | Garole |
| EF056427 | FJ218041 | isolateGa_54 | Hap_145 | India | A | Asia | India | Garole |
| EF056428 | FJ218049 | isolateGa_689 | Hap_146 | India | A | Asia | India | Garole |
| EF056433 | FJ218092 | isolateLo_27805 | Hap_147 | India | A | Asia | India | Deccani |
| EF056434 | FJ218094 | isolateLo_27811 | Hap_148 | India | A | Asia | India | Deccani |
| EF056437 | FJ218083 | isolateLo_308 | Hap_149 | India | A | Asia | India | Deccani |
| EF056440 | FJ218084 | isolateLo_326 | Hap_150 | India | A | Asia | India | Deccani |
| EF056442 | FJ218085 | isolateLo_349 | Hap_151 | India | A | Asia | India | Deccani |
| EF056443 | FJ218086 | isolateLo_354 | Hap_152 | India | A | Asia | India | Deccani |
| EF056446 | FJ218089 | isolateLo_656 | Hap_153 | India | A | Asia | India | Deccani |
| EF056447 | FJ218091 | isolateLo_696 | Hap_154 | India | A | Asia | India | Deccani |
| EF056453 | FJ218128 | isolateSn_2932 | Hap_155 | India | A | Asia | India | Deccani |
| EF056459 | FJ218143 | isolateSn_3621 | Hap_156 | India | A | Asia | India | Deccani |
| EF056462 | FJ218146 | isolateSn_3628 | Hap_157 | India | A | Asia | India | Deccani |
| EF056450 | FJ218126 | isolateSn_2870 | Hap_158 | India | A | Asia | India | Deccani |
| EF056414 | FJ218038 | isolateGa_1 | Hap_159 | India | A | Asia | India | Garole |
| EF056465 | FJ218148 | isolateSn_3648 | Hap_160 | India | A | Asia | India | Deccani |
| EF056457 | FJ218139 | isolateSn_3612 | Hap_161 | India | A | Asia | India | Deccani |
|  |  |  |  |  |  |  |  |  |
