# Supplementary material for: Phylogenetic Position of a Copper Age Sheep (Ovis aries) Mitochondrial DNA
Source: PLoS One. 2012 Mar 23;7(3):e33792. doi: 10.1371/journal.pone.0033792 (PMC3311544; doi:10.1371/journal.pone.0033792)
Supplement: Table S6 — Distribution of haplogroups (in percentage) within different geographic area. The outgroup sequence Ovis Vignei is excluded. *Paraphyletic group. (DOC) [file pone.0033792.s009.doc]

**Table S6. Distribution of haplogroups (in percentage) within different geographic area. The outgroup sequence *Ovis Vignei* is excluded. *Paraphyletic group.**

|  | **A** | **B** | **B*** | **C** | **E** | **D*** |
| --- | --- | --- | --- | --- | --- | --- |
| **Southern Central Asia** | 75 | 25 | 0 | 0 | 0 | 0 |
| **Europe** | 15 | 79 | 6 | 0 | 0 | 0 |
| **Middle East** | 20 | 60 | 4 | 13 | 2 | 1 |
| **Oceania** | 25 | 75 | 0 | 0 | 0 | 0 |
